# The Associations of Anthropometric Indices With Stages and Mortality in Cardiovascular–Kidney–Metabolic Syndrome: Insights From NHANES

**DOI:** 10.31083/RCM46650

**Published:** 2026-02-25

**Authors:** Ming Zhong, Chen-nan Liu, Yang Chen

**Affiliations:** ^1^Department of Endocrinology, The First Affiliated Hospital of Fujian Medical University, Fujian Medical University, 350005 Fuzhou, Fujian, China; ^2^Department of Endocrinology, National Regional Medical Center, Binhai Campus of the First Affiliated Hospital of Fujian Medical University, Fujian Medical University, 350212 Fuzhou, Fujian, China; ^3^Clinical Research Center for Metabolic Diseases of Fujian Province, The First Affiliated Hospital of Fujian Medical University, Fujian Medical University, 350005 Fuzhou, Fujian, China; ^4^Fujian Key Laboratory of Glycolipid and Bone Mineral Metabolism, The First Affiliated Hospital of Fujian Medical University, Fujian Medical University, 350005 Fuzhou, Fujian, China; ^5^Diabetes Research Institute of Fujian Province, The First Affiliated Hospital of Fujian Medical University, Fujian Medical University, 350005 Fuzhou, Fujian, China; ^6^Department of Cardiology, The First Affiliated Hospital of Fujian Medical University, Fujian Medical University, 350005 Fuzhou, Fujian, China; ^7^Department of Cardiology, National Regional Medical Center, The First Affiliated Hospital of Fujian Medical University, Fujian Medical University, 350212 Fuzhou, Fujian, China; ^8^Department of Cardiovascular and Metabolic Medicine, Institute of Life Course and Medical Sciences, University of Liverpool, L7 8TX Liverpool, UK; ^9^Liverpool Centre for Cardiovascular Science at University of Liverpool, Liverpool John Moores University and Liverpool Heart and Chest Hospital, L7 8TX Liverpool, UK

**Keywords:** anthropometry, cardio-renal syndrome, metabolic syndrome, mortality, disease progression

## Abstract

**Background:**

Cardiovascular–kidney–metabolic (CKM) syndrome embodies the interconnection between cardiovascular, renal, and metabolic disorders. Anthropometric indices reflect distinct aspects of obesity and may aid in stratifying the severity of CKM syndrome and predicting mortality. Thus, this study aimed to assess and compare the relationships between multiple obesity-related measures and advanced CKM stages, as well as the risk of mortality.

**Methods:**

Data included in this analysis were from the National Health and Nutrition Examination Survey (NHANES). Participants were categorized into quartiles (Q1–Q4) based on each anthropometric index. We estimated the associations with all-cause, cardiovascular, and non-cardiovascular mortality outcomes using Cox proportional hazards models, and evaluated the odds of an advanced CKM stage (stages 3/4) using logistic regression. Possible non-linear exposure–outcome patterns were further investigated through restricted cubic spline modelling. Then, to compare the predictive performance of the indices, we calculated the area under the receiver operating characteristic curve (AUC).

**Results:**

We included 28,911 adults from the NHANES (1999–2018) (median age (interquartile range (IQR)) 55.0 (40.0–67.0) years, 52.5% male), comprising 21,789 in CKM stages 1–2 and 7122 in stages 3–4. The anthropometric indices varied significantly across CKM stages (*p* < 0.001), with body mass index, waist circumference, Weight-adjusted Waist Index (WWI), and relative fat mass increasing with disease severity. In stages 1–2, the highest quartile (Q4) of A Body Shape Index (ABSI), WWI, waist-to-height ratio (WHtR), and Conicity Index (C-index) was associated with higher all-cause and cardiovascular mortalities, often following U-shaped or J-shaped non-linear patterns. In stages 3–4, predictive strength diminished, with only the ABSI and WWI showing consistent associations with mortality. For CKM progression, the ABSI (AUC = 0.73), WWI (AUC = 0.70), and C-index (AUC = 0.69) demonstrated the best discrimination.

**Conclusions:**

This study shows that several anthropometric indices, particularly the ABSI, WWI, WHtR, and C-index, are strongly associated with advanced CKM stage and increased mortality risk. These associations were stronger for central adiposity measures than for general adiposity, suggesting the potential relevance of central fat distribution and supporting the possible role of anthropometric indices in early risk stratification and targeted intervention in CKM syndrome.

## 1. Introduction

Cardiovascular-kidney-metabolic (CKM) syndrome is a new medical concept introduced and officially defined by the American Heart Association (AHA) in 2023. It aims to emphasize the close connections, mutual influences, and shared pathophysiological basis between metabolic disorders, chronic kidney disease (CKD), and cardiovascular diseases (CVD) (such as obesity, type 2 diabetes, and metabolic dysfunction-related fatty liver disease) [1]. The global pandemics of obesity, prediabetes/diabetes, and population aging signal that the prevalence of CKM syndrome will continue to rise [1, 2]. CKM syndrome is a major cause of premature death, disability, and skyrocketing healthcare costs. It represents one of the most significant challenges currently facing global public health [3].

CKM syndrome refers to a comprehensive condition where CKD, metabolic risk factors, and metabolic disorders (especially obesity and insulin resistance) collectively elevate the risk of CVD [1]. Its core mechanism lies in excess adiposity, which drives chronic low-grade inflammation and insulin resistance, establishing a reinforcing cycle that leads to progressive cardiac, renal, and vascular injury [4]. Over 90% of adults will experience overweight or obesity during their lifetime, and most of them will have at least one metabolic abnormality or target organ damage, indicating that the majority of overweight/obese individuals are at risk for CKM syndrome [5]. Systemic obesity (body mass index [BMI], A Body Shape Index [ABSI], and Weight-adjusted Waist Index [WWI]) can trigger insulin resistance, hyperglycemia, and dyslipidemia, further promoting the development of atherosclerosis and CKD [6, 7, 8]. Visceral fat (lipid accumulation product [LAP] and visceral adiposity index [VAI]) secretes pro-inflammatory factors (such as TNF-α, IL-6) and abnormal adipokines (e.g., elevated leptin, reduced adiponectin), which directly worsen insulin resistance [9, 10]. Abdominal fat (waist circumference [WC], waist-to-height ratio [WHtR], and waist-to-hip ratio [WHR]) accumulation is an independent predictor of cardiovascular events [11]. Its mechanisms include myocardial fibrosis inducing arrhythmias and left ventricular hypertrophy leading to heart failure [4, 12]. Furthermore, central obesity can increase intra-abdominal pressure, compress the renal veins, causing proteinuria, and increase cardiac afterload [13]. Sarcopenic obesity reduces (relative fat mass [RFM] and body adiposity index [BAI]) metabolic flexibility, amplifies insulin resistance, and increases the risk of frailty and infections [14, 15, 16]. Morphological and volume-related indicators (abdominal volume index [AVI], C-index, and Body Roundness Index [BRI]) promote oxidative stress, while increasing cardiac load, increasing heart failure-related mortality, and triggering multiple organ failure [17]. These factors work together to drive the progression of CKM syndrome, leading to multi-organ dysfunction and ultimately resulting in cardiovascular or non-cardiovascular related mortality.

Research on predicting the prognosis of CKM has developed various risk assessment models. The high-sensitivity C-reactive protein/high-density lipoprotein cholesterol (HDL-C) ratio is closely linked to all-cause mortality risk in CKM patients [18], while the estimated glucose disposal rate (eGDR) effectively predicts prognosis by assessing insulin resistance [19]. Additionally, models incorporating social factors, like those from the China Health and Retirement Longitudinal Study (CHARLS), highlight the impact of the social environment on mental health [20]. Machine learning techniques, such as random forests and XGBoost, improve prediction accuracy [21]. These advancements offer valuable insights for clinical management. Growing research attention has been directed toward newly proposed obesity- and lipid-related indices, as accumulating evidence suggests that they may be closely associated with CKM. The AHA classifies CKM into stages from CKM 0 to CKM 4, with CKM stages 1 and 2 involving multiple metabolic risk factors but no organ damage [1]. Stages 3 and 4 of CKM are characterized by structural organ damage and functional failure, with obesity shifting to sarcopenic obesity and central fat accumulation. The disease is often irreversible at this stage [22, 23]. Elevated BMI, WC, and BRI are linked to a higher risk of cardiovascular mortality [24, 25, 26]. A higher metabolic score for visceral fat (METS-VF) is related to higher risks of CVD and all-cause mortality in individuals with varying glycaemic status [27]. A nationwide longitudinal study reported that abdominal obesity, as assessed by the BRI, is closely related to the progression of frailty across different stages of the CKM syndrome [28].

Given the substantial differences in mortality risk between CKM stages 1–2 and 3–4 reported in a previous publication [29], it is essential to assess prognostic markers within early and advanced stages separately. Therefore, this study aimed to utilise data from National Health and Nutrition Examination Survey (NHANES) to (1) compare the associations of multiple anthropometric indices with all-cause and cause-specific mortality in patients with CKM stages 1–2 and 3–4, respectively; and (2) compare the cross-sectional associations of multiple anthropometric indicators with advanced CKM staging.

## 2. Materials and Methods

### 2.1 Data Sources

This analysis used data from the NHANES, a nationally representative programme conducted by the U.S. Centers for Disease Control and Prevention to monitor population health and nutritional status. The survey protocol received approval from the National Center for Health Statistics Ethics Review Board, and written informed consent was obtained from all participants. The datasets are publicly accessible at: https://www.cdc.gov/nchs/nhanes/. The study followed the Strengthening the Reporting of Observational Studies in Epidemiology (STROBE) reporting guidelines (**Supplementary Table 1**) [30].

### 2.2 Participant Selection

A total of 55,081 adults aged ≥20 years were initially identified from ten NHANES cycles (1999–2018). We excluded participants with incomplete CKM-related variables (N = 17,949), participants without CKM (N = 1752), those with missing values for height (N = 828), weight (N = 118), or waist circumference (N = 1093), participants with missing baseline variables (N = 4388), and those with uncertain survival information (N = 42), 28,911 participants were retained for analysis (**Supplementary Fig. 1**).

### 2.3 Covariates Selection

We extracted demographic factors (age, race, sex, height, weight, WC, hip measurement), socioeconomic indicators (marital status, poverty-to-income ratio, education level), lifestyle behaviors (physical activity, smoking status, alcohol consumption), comorbid conditions (diabetes, hypertension, CKD, prior stroke, CVD), and laboratory indicators (hemoglobin A1c, high-density lipoprotein cholesterol [HDL-C], urine albumin-to-creatinine ratio [UACR]), estimated glomerular filtration rate (eGFR), total cholesterol, 10-year predicted CVD risk score, Systemic Immune-Inflammation Index (SII), and frailty score (**Supplementary Table 2**) [29].

### 2.4 Definitions of CKM

CKM staging followed the AHA classification framework and prior publications [1, 29, 31]. Participants were assigned to CKM stages 1–4 using a predefined algorithm that integrates metabolic abnormalities, kidney disease, and CVD. Metabolic abnormalities included obesity, dysglycemia, hypertension, hypertriglyceridemia, or metabolic syndrome. CVD was defined as clinical CVD (heart failure, coronary heart disease, myocardial infarction, or stroke) or elevated subclinical risk, estimated using the AHA PREVENT 10-year CVD risk equations (≥20%) [32]. Kidney disease was defined according to Kidney Disease: Improving Global Outcomes (KDIGO) criteria for reduced eGFR and/or elevated albuminuria [33]. This staging reflects progression from metabolic risk to kidney involvement and overt CVD. This staging reflects progression from metabolic risk to kidney involvement and overt CVD. Full diagnostic thresholds and component definitions are provided in **Supplementary Tables 3,4,5**. CKM stages were grouped as “early CKM” (stages 1–2) and “advanced CKM” (stages 3–4). Stages 1–2 were considered early because they are driven mainly by cardiometabolic risk factors and early organ involvement, which may still be modifiable. Stages 3–4 were considered advanced because they reflect established end-organ damage, including chronic kidney dysfunction and/or clinical CVD.

### 2.5 Assessment of Anthropometric Indices

Height, Weight, WC, and hip measurements were measured with calibrated equipment according to established protocols (https://wwwn.cdc.gov/Nchs/Data/Nhanes/Public/1999/DataFiles/BMX.htm). Anthropometric measures included BMI, WC, BRI, WWI, ABSI, RFM, WHtR, C-index, VAI, LAP, WHR, BAI, AVI were determined using specified equations (**Supplementary Table 6**).

### 2.6 Mortality Outcomes

Three mortality outcomes were considered in the analysis: all-cause mortality, cardiovascular mortality, and non-cardiovascular mortality. Vital status and causes of death were identified through the National Death Index, which is integrated with NHANES and overseen by the U.S. Centers for Disease Control and Prevention. Mortality follow-up extended until 31 December 2019. Causes of death were classified using the Tenth Revision of the International Classification of Diseases. Person-time was calculated from the date of each participant’s NHANES interview to either death or the end of follow-up.

### 2.7 Statistical Analysis

In this study, missing values were excluded from the analyses. Continuous measures were summarized using the median and interquartile ranges (IQR). Between-group comparisons for continuous variables employed nonparametric Kruskal-Wallis tests. Categorical variables were evaluated through Fisher’s exact tests or Pearson’s chi-square tests, with outcomes presented as frequency counts and proportional percentages.

Participants were classified into early CKM stage (CKM stages 1–2) and advanced CKM stage (CKM stages 3–4). For each anthropometric indicator, individuals were further grouped by quartiles (Q1–Q4). To examine mortality risk, we fitted Cox proportional hazards models for each index (BMI, WC, BRI, WWI, RFM, ABSI, WHtR, and C-index) with Q1 serving as the reference category, and reported hazard ratios (HRs) and 95% confidence intervals (CIs). The models were adjusted for a range of potential confounders, including age, sex, race, socioeconomic status (poverty income ratio, marital status, education), lifestyle factors (smoking, alcohol use, physical activity), and comorbid conditions (CVD, hypertension, diabetes, CKD, and stroke). To investigate possible non-linear dose–response relationships, restricted cubic spline (RCS) functions were applied to continuous forms of the anthropometric indices. Furthermore, the discriminatory capacity of each index for predicting mortality among individuals with CKM was assessed by receiver operating characteristic (ROC) analysis, with area under the receiver operating characteristic curve (AUC) values reported and pairwise differences compared using the DeLong test.

Then, we performed multivariable logistic regression to evaluate the relationships between anthropometric indicators and advanced CKM stage (stages 3–4 vs. 1–2), adjusting for the same covariates as in the Cox models. Odds ratios (ORs) and 95% CIs were calculated by comparing the highest quartile (Q4) with the lowest quartile (Q1) for each anthropometric index. To assess potential non-linear dose–response patterns, RCS models were applied. The predictive performance of the indices for advanced CKM stage and CKM-related mortality was evaluated using ROC curves, and AUC values were statistically compared using the DeLong test.

We conducted two sensitivity analyses: first, to address potential residual confounding, we performed additional sensitivity analyses in which we further adjusted the Cox proportional hazards models for frailty score; second, to mitigate reverse causality, we excluded patients who died within the first two years of follow-up and re-examined the impact of various obesity-related indicators on mortality among CKM stage 3–4 patients.

Additionally, patients with missing data on triglycerides (N = 12,426) and hip circumference (N = 25,897) were excluded from the respective analyses. Specifically, ROC analyses of VAI and LAP were conducted within the triglyceride-available cohort, and those of WHR, BAI, and AVI were performed within the hip circumference-available cohort. In each subset, these indices were compared against the primary anthropometric measures to evaluate relative discriminatory performance.

Statistical analyses were conducted using SPSS Statistics (version 27; IBM Corporation, Armonk, NY, USA) and R software (version 4.4.2; R Foundation for Statistical Computing, Vienna, Austria). A two-sided significance threshold of *p* < 0.05 was applied.

## 3. Results

### 3.1 Baseline Characteristics

A total of 28,911 eligible participants were included in this analysis, with a median age of 55.0 years (IQR: 40.0–67.0), 52.5% males. Table 1 presented the baseline characteristics of participants, categorized by CKM staging. The cohort consisted of 21,789 CKM stage 1–2 patients and 7122 CKM stage 3–4 patients. Anthropometric indices showed notable differences across CKM stages. BMI, WC, RFM, WWI and other adiposity-related measures were generally higher in CKM stages 2–4 compared with stage 1, although the specific pattern of change varied by index. Overall, all anthropometric indices differed significantly across CKM stages (*p* < 0.001).

**Table 1.** **Baseline characteristics based on different CKM stages**.

Characteristics		All CKM (N = 28,911)	CKM stage 1 (N = 3254)	CKM stage 2 (N = 18,535)	CKM stage 3 (N = 3336)	CKM stage 4 (N = 3786)	*p*
Age, years		55.0 (40.0, 67.0)	40.0 (30.0, 51.0)	50.0 (39.0, 61.0)	78.0 (72.0, 80.0)	68.0 (60.0, 77.0)	<0.001
Male, n (%)		15,187.0 (52.5)	1589.0 (48.8)	9408.0 (50.8)	1917.0 (57.5)	2273.0 (60.0)	<0.001
Race, n (%)							<0.001
	Mexican American	4820.0 (16.7)	618.0 (19.0)	3367.0 (18.2)	435.0 (13.0)	400.0 (10.6)	
	Non-Hispanic Black	6008.0 (20.8)	658.0 (20.2)	4029.0 (21.7)	567.0 (17.0)	754.0 (19.9)	
	Non-Hispanic White	13,372.0 (46.3)	1298.0 (39.9)	7894.0 (42.6)	1976.0 (59.2)	2204.0 (58.2)	
	Hispanic and others	4711.0 (16.3)	680.0 (20.9)	3245.0 (17.5)	358.0 (10.7)	428.0 (11.3)	
Poverty income ratio		2.2 (1.2, 4.1)	2.5 (1.2, 4.5)	2.3 (1.2, 4.3)	1.9 (1.2, 3.3)	1.8 (1.1, 3.3)	<0.001
Education, n (%)							<0.001
	College or above	14,181.0 (49.1)	1938.0 (59.6)	9395.0 (50.7)	1316.0 (39.4)	1532.0 (40.5)	
	High school or equivalent	11,159.0 (38.6)	1065.0 (32.7)	7125.0 (38.4)	1324.0 (39.7)	1645.0 (43.4)	
	Less than high school	3571.0 (12.4)	251.0 (7.7)	2015.0 (10.9)	696.0 (20.9)	609.0 (16.1)	
Marital status, n (%)							<0.001
	Unmarried	3872.0 (13.4)	749.0 (23.0)	2764.0 (14.9)	123.0 (3.7)	236.0 (6.2)	
	Married	17,804.0 (61.6)	2044.0 (62.8)	11,701.0 (63.1)	1853.0 (55.5)	2206.0 (58.3)	
	Divorced	7235.0 (25.0)	461.0 (14.2)	4070.0 (22.0)	1360.0 (40.8)	1344.0 (35.5)	
Smoking status, n (%)							<0.001
	Current smoker	5826.0 (20.2)	589.0 (18.1)	4233.0 (22.8)	243.0 (7.3)	761.0 (20.1)	
	Former smoker	8161.0 (28.2)	674.0 (20.7)	4464.0 (24.1)	1438.0 (43.1)	1585.0 (41.9)	
	Never smoker	14,924.0 (51.6)	1991.0 (61.2)	9838.0 (53.1)	1655.0 (49.6)	1440.0 (38.0)	
Alcohol consumption, n (%)							<0.001
	Heavy	3973.0 (13.7)	559.0 (17.2)	3004.0 (16.2)	118.0 (3.5)	292.0 (7.7)	
	Mild to moderate	6512.0 (22.5)	955.0 (29.3)	4628.0 (25.0)	375.0 (11.2)	554.0 (14.6)	
	Non-drinker	18,426.0 (63.7)	1740.0 (53.5)	10,903.0 (58.8)	2843.0 (85.2)	2940.0 (77.7)	
Physical activity, n (%)							<0.001
	Less than moderate	17,050.0 (59.0)	1795.0 (55.2)	10,629.0 (57.3)	2193.0 (65.7)	2433.0 (64.3)	
	Moderate	7139.0 (24.7)	763.0 (23.4)	4530.0 (24.4)	881.0 (26.4)	965.0 (25.5)	
	Vigorous	4722.0 (16.3)	696.0 (21.4)	3376.0 (18.2)	262.0 (7.9)	388.0 (10.2)	
Comorbidities							
	CVD	3786.0 (13.1)	0.0 (0.0)	0.0 (0.0)	0.0 (0.0)	3786.0 (100.0)	<0.001
	Hypertensive	16,418.0 (56.8)	0.0 (0.0)	10,683.0 (57.6)	2775.0 (83.2)	2960.0 (78.2)	<0.001
	Diabetes	6614.0 (22.9)	151.0 (4.6)	3453.0 (18.6)	1510.0 (45.3)	1500.0 (39.6)	<0.001
	CKD	6876.0 (23.8)	0.0 (0.0)	3250.0 (17.5)	1902.0 (57.0)	1724.0 (45.5)	<0.001
	Stroke	1352.0 (4.7)	0.0 (0.0)	0.0 (0.0)	0.0 (0.0)	1352.0 (35.7)	<0.001
Laboratory indicators							
	Total cholesterol, mg/dL	195.0 (169.0, 224.0)	191.0 (166.0, 214.0)	200.0 (174.0, 229.0)	190.0 (164.0, 217.0)	179.0 (152.0, 211.0)	<0.001
	HDL-C, mg/dL	50.0 (41.0, 61.0)	57.0 (51.0, 66.0)	48.0 (40.0, 60.0)	49.0 (41.0, 60.0)	47.0 (39.0, 58.0)	<0.001
	eGFR, mL/min/1.73 m^2^	91.1 (74.1, 106.0)	104.3 (91.0, 116.8)	95.6 (81.6, 108.6)	64.4 (50.3, 79.3)	73.3 (55.6, 88.7)	<0.001
	UACR, mg/g	7.7 (4.7, 17.0)	5.2 (3.8, 7.8)	7.3 (4.7, 14.6)	14.7 (7.4, 42.0)	11.5 (6.2, 34.7)	<0.001
	Hemoglobin A1c	5.6 (5.3, 6.0)	5.4 (5.1, 5.6)	5.5 (5.3, 5.9)	5.9 (5.5, 6.6)	5.8 (5.5, 6.4)	<0.001
SII		476.1 (338.5, 669.4)	430.4 (310.2, 598.5)	473.5 (340.0, 660.2)	502.0 (355.2, 719.2)	505.4 (344.6, 724.0)	<0.001
10-year CVD risk score		5.8 (1.6, 15.3)	1.0 (0.4, 2.8)	4.1 (1.4, 9.6)	26.0 (22.7, 30.7)	18.3 (9.4, 27.6)	<0.001
Frailty score		0.1 (0.1, 0.2)	0.1 (0.1, 0.1)	0.1 (0.1, 0.2)	0.2 (0.1, 0.2)	0.3 (0.2, 0.3)	<0.001
Anthropometric indices							
	BMI, kg/m^2^	28.7 (25.3, 33.1)	27.3 (25.1, 30.7)	29.1 (25.5, 33.7)	27.9 (24.7, 31.5)	29.0 (25.6, 33.4)	<0.001
	WC, cm	100.4 (91.1, 110.7)	94.0 (87.3, 102.7)	100.5 (91.0, 111.0)	102.0 (93.3, 110.8)	104.1 (95.2, 114.5)	<0.001
	BRI	–1.2 (–1.2, –1.1)	–1.2 (–1.2, –1.2)	–1.2 (–1.2, –1.1)	–1.2 (–1.2, –1.1)	–1.2 (–1.2, –1.1)	<0.001
	WWI	11.2 (10.6, 11.7)	10.7 (10.2, 11.2)	11.1 (10.6, 11.6)	11.7 (11.2, 12.1)	11.5 (11.1, 12.0)	<0.001
	ABSI	0.1 (0.1, 0.1)	0.1 (0.1, 0.1)	0.1 (0.1, 0.1)	0.1 (0.1, 0.1)	0.1 (0.1, 0.1)	<0.001
	RFM, %	35.2 (29.6, 43.3)	34.8 (27.6, 41.3)	35.5 (29.5, 43.7)	34.6 (30.6, 43.1)	35.0 (30.4, 43.4)	<0.001
	WHR	0.6 (0.5, 0.7)	0.6 (0.5, 0.6)	0.6 (0.5, 0.7)	0.6 (0.6, 0.7)	0.6 (0.6, 0.7)	<0.001
	C-Index	1.3 (1.3, 1.4)	1.3 (1.2, 1.3)	1.3 (1.3, 1.4)	1.4 (1.3, 1.4)	1.4 (1.3, 1.4)	<0.001

Values are expressed as the median (interquartile range) or percentage (%). Abbreviations: ABSI, A Body Shape Index; BMI, body mass index; BRI, Body Roundness Index; C-index, Conicity Index; CKD, chronic kidney disease; CKM, cardiovascular-kidney-metabolic syndrome; CVD, cardiovascular disease; eGFR, estimated glomerular filtration rate; HDL-C, high-density lipoprotein cholesterol; RFM, relative fat mass; UACR, urinary albumin to creatinine ratio; SII, Systemic Immune-Inflammation Index; WC, waist circumference; WHR, waist-to-height ratio; WWI, Weight-adjusted Waist Index.

Participants in CKM stages 1–2 had comparatively low mortality, with 8.7% dying from all causes, 1.8% from cardiovascular disease, and 6.9% from non-cardiovascular causes. In contrast, mortality was substantially higher among those in CKM stages 3–4, with 45.9% all-cause, 14.1% cardiovascular, and 31.8% non-cardiovascular deaths (**Supplementary Fig. 2**).

### 3.2 Relationship Between Anthropometric Indices and Mortality Outcomes in CKM Stage 1–2 Patients

Table 2 presents the associations between anthropometric indices and mortality outcomes among patients with CKM stages 1–2. Q4 of BMI was linked to increased cardiovascular mortality (HR 1.41, *p* = 0.020) and lower non-cardiovascular mortality (HR 0.78, *p* = 0.002). Q4 of WC, BRI, RFM, and WHtR were linked to increased cardiovascular mortality (HR 1.39 vs. 1.42 vs. 1.73 vs. 1.56, all *p* < 0.05). Q4 of WWI and ABSI were linked to increased all-cause mortality (HR 1.62 vs. 1.43, all *p* < 0.05), cardiovascular mortality (HR 1.38 vs. 1.62, all *p* < 0.05), and non-cardiovascular mortality (HR 1.16 vs. 1.38, all *p* < 0.05). Q4 of C-index was linked to increased all-cause mortality (HR 1.18, *p* = 0.018) and cardiovascular mortality (HR 1.56, *p* = 0.005).

**Table 2.** **The associations between anthropometric indices and mortality outcomes in CKM stage 1–2 patients**.

		All-cause mortality		Cardiovascular mortality		Non-cardiovascular mortality	
		HR (95% CI)	*p*	HR (95% CI)	*p*	HR (95% CI)	*p*
BMI							
	Q1	Reference		Reference		Reference	
	Q2	0.79 (0.69, 0.89)	<0.001	1.09 (0.77, 1.45)	0.553	0.73 (0.64, 0.85)	<0.001
	Q3	0.81 (0.71, 0.92)	0.001	1.04 (0.77, 1.40)	0.802	0.77 (0.66, 0.88)	<0.001
	Q4	0.88 (0.77, 1.01)	0.079	1.41 (1.05, 1.89)	0.020	0.78 (0.68, 0.91)	0.002
WC							
	Q1	Reference		Reference		Reference	
	Q2	0.86 (0.76, 0.98)	0.030	0.88 (0.65, 1.19)	0.416	0.86 (0.75, 0.99)	0.045
	Q3	0.83 (0.73, 0.95)	0.008	0.97 (0.72, 1.32)	0.874	0.80 (0.69, 0.94)	0.004
	Q4	0.99 (0.87, 1.13)	0.920	1.39 (1.04, 1.85)	0.024	0.90 (0.77, 1.05)	0.192
BRI							
	Q1	Reference		Reference		Reference	
	Q2	0.81 (0.71, 0.92)	0.001	0.95 (0.71, 1.28)	0.747	0.78 (0.67, 0.90)	<0.001
	Q3	0.82 (0.72, 0.94)	0.005	0.89 (0.65, 1.22)	0.487	0.81 (0.70, 0.94)	0.005
	Q4	0.96 (0.84, 1.10)	0.589	1.42 (1.06, 1.92)	0.019	0.86 (0.74, 1.10)	0.064
WWI							
	Q1	Reference		Reference		Reference	
	Q2	1.09 (0.78, 1.51)	0.611	1.00 (0.85, 1.18)	0.973	0.94 (0.80, 1.09)	0.426
	Q3	1.56 (0.83, 1.60)	0.385	1.15 (0.98, 1.36)	0.077	1.05 (0.90, 1.23)	0.493
	Q4	1.62 (1.16, 2.25)	0.004	1.38 (1.17, 1.63)	<0.001	1.16 (1.09, 1.37)	0.040
ABSI							
	Q1	Reference		Reference		Reference	
	Q2	1.02 (0.87, 1.18)	0.793	1.09 (0.78, 1.52)	0.611	1.00 (0.84, 1.18)	0.973
	Q3	1.16 (1.01, 1.34)	0.047	1.15 (0.83, 1.60)	0.385	1.15 (0.98, 1.36)	0.077
	Q4	1.43 (1.23, 1.65)	<0.001	1.62 (1.16, 2.25)	0.004	1.38 (1.17, 1.63)	<0.001
RFM							
	Q1	Reference		Reference		Reference	
	Q2	0.94 (0.82, 1.07)	0.376	1.11 (0.82, 1.47)	0.491	0.90 (0.77, 1.04)	0.163
	Q3	0.94 (0.78, 1.13)	0.540	1.47 (0.98, 2.19)	0.057	0.83 (0.67, 1.03)	0.091
	Q4	0.98 (0.78, 1.21)	0.857	1.73 (1.07, 2.80)	0.025	0.84 (0.66, 1.07)	0.174
WHtR							
	Q1	Reference		Reference		Reference	
	Q2	0.84 (0.74, 0.96)	0.011	1.12 (0.82, 1.51)	0.465	0.79 (0.68, 0.91)	0.002
	Q3	0.92 (0.81, 1.05)	0.222	1.24 (0.92, 1.68)	0.155	0.86 (0.75, 1.00)	0.043
	Q4	1.03 (0.89, 1.17)	0.709	1.56 (1.56, 2.11)	0.004	0.92 (0.86, 0.75)	0.313
C-index							
	Q1	Reference		Reference		Reference	
	Q2	0.89 (0.77, 1.03)	0.110	0.93 (0.67, 1.29)	0.670	0.88 (0.75, 1.03)	0.115
	Q3	1.00 (0.87, 1.15)	0.970	1.13 (0.83, 1.55)	0.423	0.97 (0.83, 1.13)	0.717
	Q4	1.18 (1.03, 1.35)	0.018	1.56 (1.14, 2.13)	0.005	1.10 (0.94, 1.28)	0.235

Cox proportional hazards model was adjusted for age, sex, race and ethnicity, poverty income ratio, marital status, education, smoking status, alcohol consumption, physical activity, cardiovascular disease, hypertensive, diabetes, chronic kidney disease, stroke. Abbreviations: ABSI, A Body Shape Index; BMI, body mass index; BRI, Body Roundness Index; CI, confidence interval; C-index, Conicity Index; CKM, cardiovascular-kidney-metabolic syndrome; HR, hazard ratio; RFM, relative fat mass; WC, waist circumference; WHtR, waist-to-height ratio; WWI, Weight-adjusted Waist Index.

**Fig. 1.**
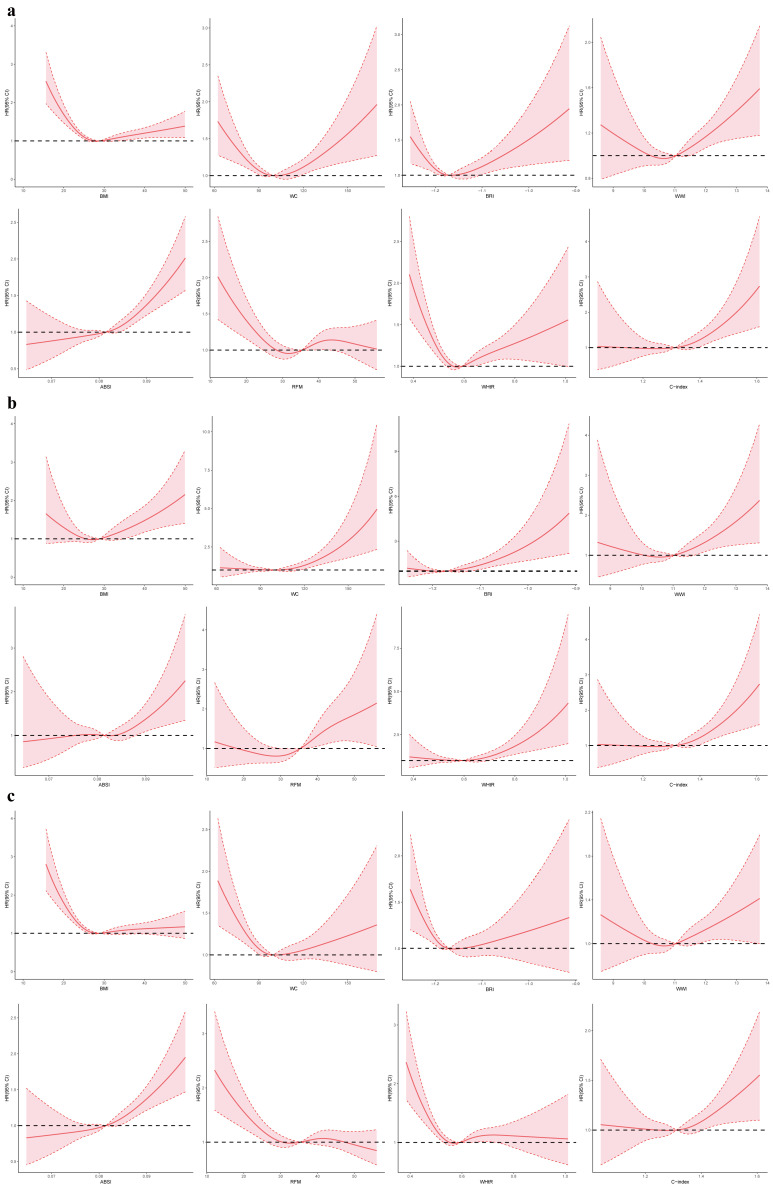
**Restricted cubic spline modelling of the association between anthropometric indices and mortality among individuals with CKM stages 1–2**. (a–c) correspond to all-cause, cardiovascular, and non-cardiovascular mortality, respectively. HR, hazard ratio; CI, confidence interval; CKM, cardiovascular-kidney-metabolic syndrome; ABSI, A Body Shape Index; BMI, body mass index; BRI, Body Roundness Index; C-index, Conicity Index; RFM, relative fat mass; WC, waist circumference; WHtR, waist-to-height ratio; WWI, Weight-adjusted Waist Index.

RCS revealed non-linear associations between anthropometric indices and mortality outcomes in CKM stages 1–2 patients (Fig. 1). For all-cause mortality (Fig. 1a), BMI, WHtR, BRI, and C-index showed U-shaped relationships, with the lowest risk observed at a BMI of 25–27 kg/m^2^ and a WHtR of around 0.5. In contrast, WC and WWI demonstrated J-shaped curves, indicating a continuous increase in risk with greater central adiposity. For cardiovascular mortality (Fig. 1b), WC, WHtR, BRI, and C-index displayed steep J-shaped patterns, with risk rising sharply at higher values. For non-cardiovascular mortality (Fig. 1c), BMI retained a U-shaped association, with elevated risk at BMI <20 kg/m^2^, whereas WC, WWI, and RFM showed only mild or nonsignificant relationships.

### 3.3 Relationship Between Anthropometric Indices and Mortality Outcomes in CKM Stage 3–4 Patients

Anthropometric indices showed significant associations with mortality outcomes in CKM stages 3–4 (Table 3). Q4 of BMI, WC, BRI, and WHtR were linked to decreased all-cause mortality (HR 0.87 vs. 0.87 vs. 0.84 vs. 0.86, all *p* < 0.05) and non-cardiovascular mortality (HR 0.84 vs. 0.85 vs. 0.81 vs. 0.81, all *p* < 0.05). Q4 of WWI was linked to increased cardiovascular mortality (HR 1.35, *p* = 0.029). Q4 of ABSI was linked to increased all-cause mortality (HR 1.18, *p* = 0.022) and cardiovascular mortality (HR 1.41, *p* = 0.019). Q4 of RFM was linked to increased non-cardiovascular mortality (HR 1.04, *p* = 0.046). Q2 of C-index was linked to decreased all-cause mortality (HR 0.81, *p* = 0.011) and non-cardiovascular mortality (HR 0.86, *p* = 0.037).

**Table 3.** **The associations between anthropometric indices and mortality outcomes in CKM stage 3–4 patients**.

		All-cause death		Cardiovascular death		Non-cardiovascular death	
		HR (95% CI)	*p*	HR (95% CI)	*p*	HR (95% CI)	*p*
BMI							
	Q1	Reference		Reference		Reference	
	Q2	0.86 (0.78, 0.94)	0.002	0.94 (0.90, 1.11)	0.490	0.83 (0.74, 0.93)	<0.001
	Q3	0.78 (0.71, 0.86)	<0.001	0.85 (0.71, 1.01)	0.062	0.76 (0.67, 0.85)	<0.001
	Q4	0.87 (0.78, 0.98)	0.021	0.95 (0.78, 1.16)	0.658	0.84 (0.74, 0.96)	0.014
WC							
	Q1	Reference		Reference		Reference	
	Q2	0.84 (0.75, 0.93)	<0.001	0.89 (0.74, 1.08)	0.245	0.82 (0.71, 0.92)	<0.001
	Q3	0.77 (0.69, 0.85)	<0.001	0.90 (0.75, 1.09)	0.297	0.71 (0.63, 0.81)	<0.001
	Q4	0.87 (0.78, 0.97)	0.014	0.93 (0.77, 1.14)	0.530	0.85 (0.74, 0.96)	0.012
BRI							
	Q1	Reference		Reference		Reference	
	Q2	0.78 (0.70, 0.86)	<0.001	0.85 (0.71, 1.03)	0.102	0.75 (0.60, 0.85)	<0.001
	Q3	0.74 (0.66, 0.82)	<0.001	0.84 (0.69, 1.02)	0.078	0.70 (0.62, 0.93)	<0.001
	Q4	0.84 (0.75, 0.94)	0.002	0.90 (0.73, 1.11)	0.355	0.81 (0.71, 0.93)	0.003
WWI							
	Q1	Reference		Reference		Reference	
	Q2	0.96 (0.84, 1.12)	0.639	1.32 (0.99, 1.76)	0.051	0.85 (0.72, 1.01)	0.065
	Q3	0.95 (0.82, 1.08)	0.425	1.24 (0.95, 1.63)	0.118	0.85 (0.73, 1.00)	0.050
	Q4	1.01 (0.88, 1.16)	0.832	1.35 (1.03, 1.76)	0.029	0.91 (0.78, 1.06)	0.242
ABSI							
	Q1	Reference		Reference		Reference	
	Q2	0.98 (0.84, 1.16)	0.870	1.19 (0.87, 1.64)	0.262	0.91 (0.75, 1.11)	0.365
	Q3	1.05 (1.03, 1.38)	0.493	1.33 (0.98, 1.78)	0.062	0.96 (0.80, 1.15)	0.679
	Q4	1.18 (1.03, 1.38)	0.022	1.41 (1.06, 1.89)	0.019	1.11 (0.94, 1.32)	0.219
RFM							
	Q1	Reference		Reference		Reference	
	Q2	0.97 (0.88, 1.07)	0.562	1.07 (0.90, 1.27)	0.426	0.93 (0.83, 1.05)	0.226
	Q3	1.03 (0.89, 1.18)	0.691	1.03 (0.79, 1.33)	0.817	1.03 (0.86, 1.22)	0.737
	Q4	0.84 (0.71, 1.00)	0.054	0.91 (0.66, 1.25)	0.563	1.04 (1.01, 1.06)	0.046
WHtR							
	Q1	Reference		Reference		Reference	
	Q2	0.84 (0.75, 0.93)	<0.001	0.95 (0.78, 1.16)	0.663	0.78 (0.69, 0.89)	<0.001
	Q3	0.82 (0.74, 0.90)	<0.001	0.91 (0.75, 1.10)	0.350	0.78 (0.69, 0.88)	<0.001
	Q4	0.86 (0.77, 0.96)	0.007	1.00 (0.82, 1.21)	0.998	0.81 (0.71, 0.92)	<0.001
C-index							
	Q1	Reference		Reference		Reference	
	Q2	0.81 (0.69, 0.95)	0.011	1.02 (0.78, 1.33)	0.893	0.86 (0.75, 0.99)	0.037
	Q3	0.76 (0.65, 0.88)	<0.001	1.15 (0.89, 1.46)	0.273	0.86 (0.76, 0.98)	0.023
	Q4	0.89 (0.77, 1.04)	0.148	1.09 (0.86, 1.39)	0.460	0.94 (0.84, 1.07)	0.398

Cox proportional hazards model was adjusted for age, sex, race and ethnicity, poverty income ratio, marital status, education, smoking status, alcohol consumption, physical activity, cardiovascular disease, hypertensive, diabetes, chronic kidney disease, stroke. Abbreviations: ABSI, A Body Shape Index; BMI, body mass index; BRI, Body Roundness Index; C-index, Conicity Index; CI, confidence interval; CKM, cardiovascular-kidney-metabolic syndrome; HR, hazard ratio; RFM, relative fat mass; WC, waist circumference; WHtR, waist-to-height ratio; WWI, Weight-adjusted Waist Index.

**Fig. 2.**
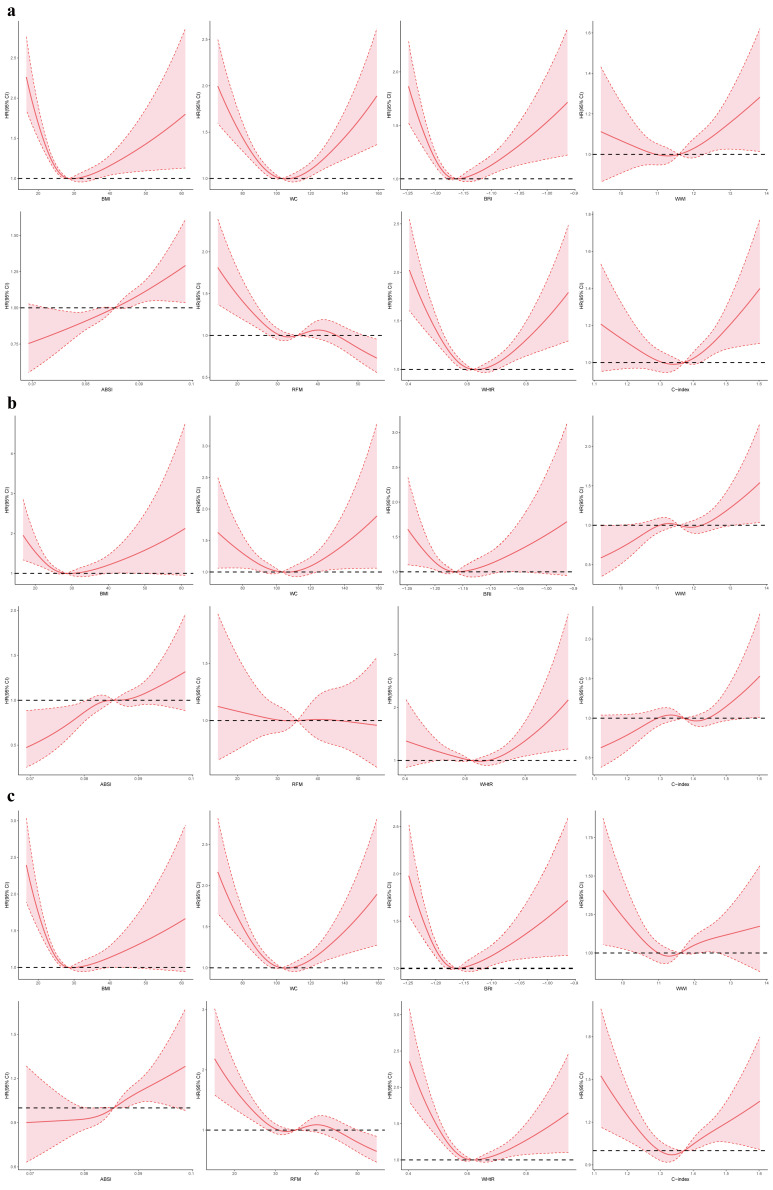
**Restricted cubic spline modelling of the association between anthropometric indices and mortality among individuals with CKM stages 3–4**. (a–c) correspond to all-cause, cardiovascular, and non-cardiovascular mortality, respectively. HR, hazard ratio; CI, confidence interval; CKM, cardiovascular-kidney-metabolic syndrome; ABSI, A Body Shape Index; BMI, body mass index; BRI, Body Roundness Index; C-index, Conicity Index; RFM, relative fat mass; WC, waist circumference; WHtR, waist-to-height ratio; WWI, Weight-adjusted Waist Index.

RCS demonstrated non-linear associations between anthropometric indices and mortality outcomes in CKM stages 3–4 patients (Fig. 2). For all-cause mortality (Fig. 2a), BMI, WC, BRI, WWI, WHtR, and C-index showed U-shaped relationships, with the lowest risk observed at a BMI of 25–27 kg/m^2^, WC of 95–105 cm, and WHtR around 0.5. In contrast, ABSI exhibited a J-shaped curve, indicating a continuous adverse effect of central adiposity. For cardiovascular mortality (Fig. 2b), BMI, WC, BRI, and WHtR displayed U-shaped patterns, whereas WWI, ABSI, and C-index followed J-shaped trends. For non-cardiovascular mortality (Fig. 2c), BMI, WC, BRI, WWI, WHtR, and C-index again showed U-shaped associations, while ABSI exhibited a J-shaped curve and RFM remained largely flat.

### 3.4 Association Between Anthropometric Indices and Advanced CKM Stage

Several anthropometric indices were significantly associated with advanced CKM stage. Participants in the highest quartile (Q4) of BMI, WC, WWI, RFM, WHtR, and C-index had increased odds of advanced CKM (ORs: 1.19, 1.98, 1.34, 1.54, 1.35, and 1.30, respectively; all *p* < 0.05; **Supplementary Table 7**). In contrast, BRI and ABSI were not significantly associated.

**Fig. 3.**
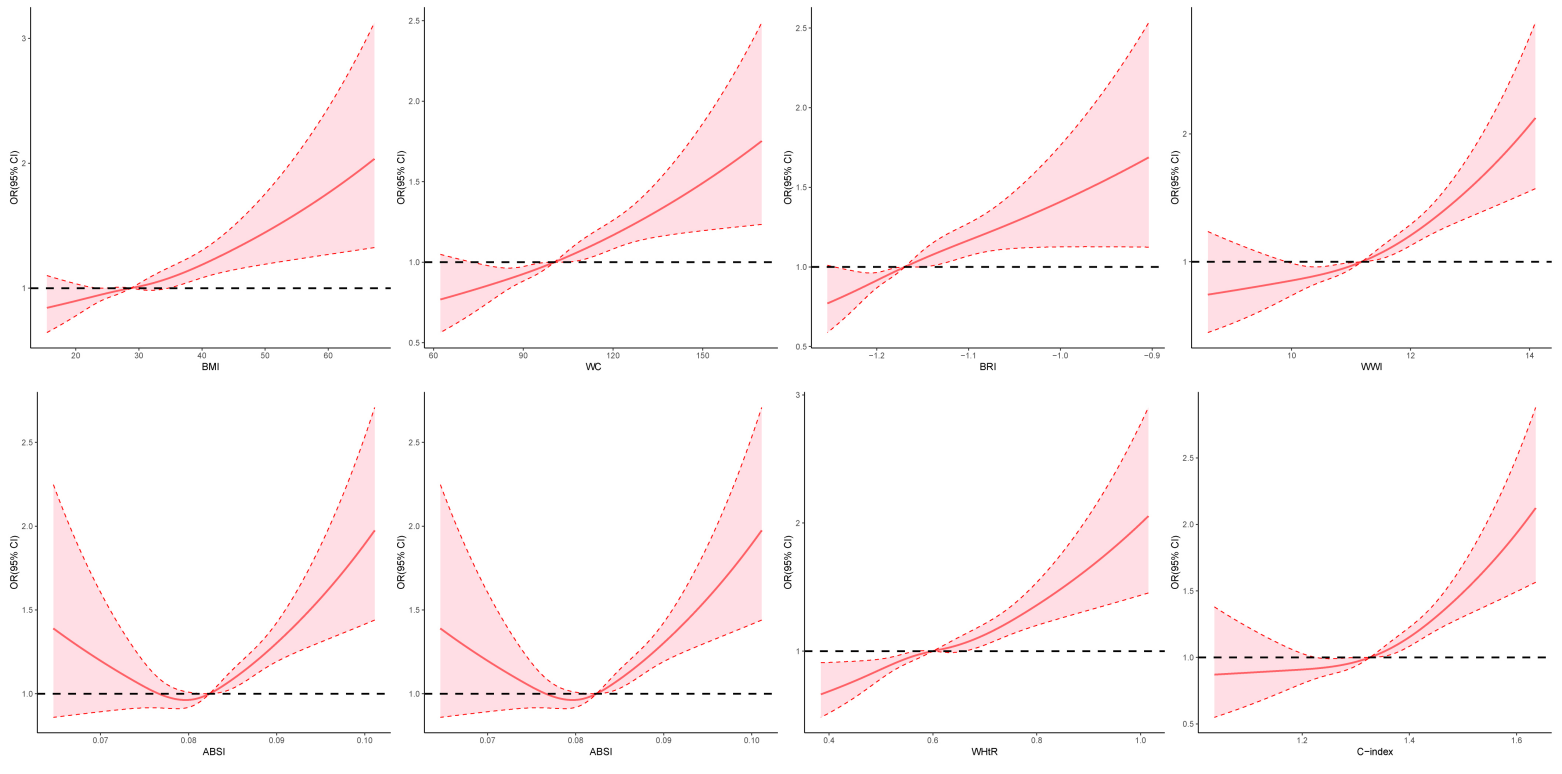
**Restricted cubic spline modeling of the associations between anthropometric indices and CKM progression**. ABSI, A Body Shape Index; BMI, body mass index; BRI, Body Roundness Index; C-index, Conicity Index; CI, confidence interval; CKM, cardiovascular-kidney-metabolic syndrome; OR, odds ratio; RFM, relative fat mass; WC, waist circumference; WHtR, waist-to-height ratio; WWI, Weight-adjusted Waist Index.

RCS analysis revealed non-linear relationships between anthropometric indices and advanced CKM stage (Fig. 3). A J-shaped pattern was observed for BMI, WC, BRI, WWI, WHtR, and C-index, indicating progressively increased risk with higher central adiposity. ABSI and RFM displayed a U-shaped association.

### 3.5 Predictive Efficacy of Anthropometric Indices for Mortality Outcomes

**Fig. 4.**
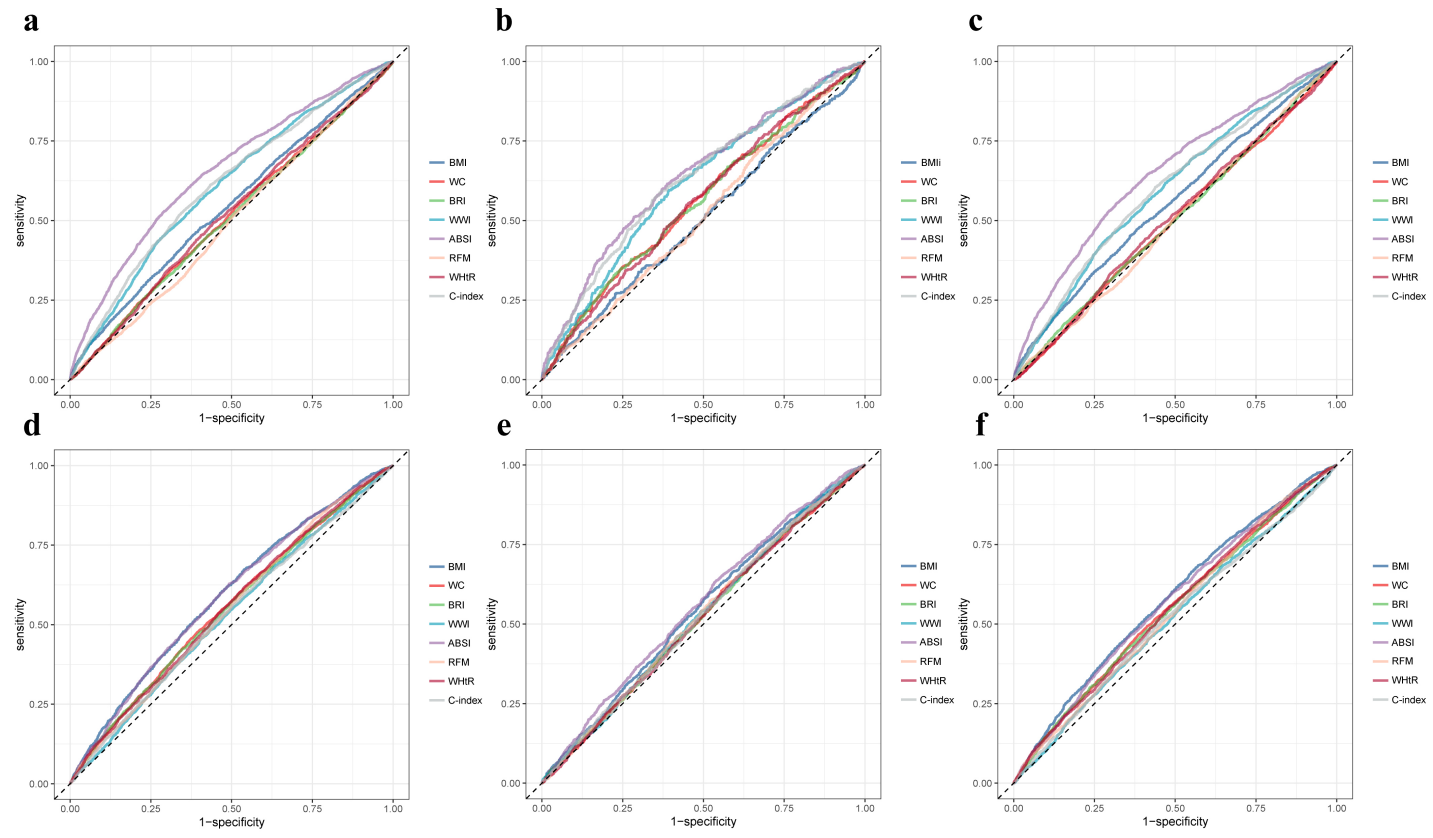
**Receiver operating characteristic curves for associations between anthropometric indices and mortality outcomes in CKM stage 1–2 or 3–4 patients**. (a–c) correspond to CKM stage 1–2, and (d–f) correspond to CKM stage 3–4, showing all-cause, cardiovascular, and non-cardiovascular mortality, respectively. ABSI, A Body Shape Index; BMI, body mass index; BRI, Body Roundness Index; C-index, Conicity Index; RFM, relative fat mass; WC, waist circumference; WWI, Weight-adjusted Waist Index; WHtR, waist-to-height ratio.

The predictive efficacy of various anthropometric indices for mortality outcomes in CKM stage 1–2 patients (**Supplementary Table 8**) showed that ABSI demonstrates the highest predictive power for all-cause, cardiovascular, and non-cardiovascular mortalities (AUC 0.65 vs. 0.64 vs. 0.65). While most of the other indices (BMI, WC, BRI, WWI, RFM, WHtR, C-index, VAI, LAP, WHR) are significantly associated with mortality outcomes, BAI and AVI showed no statistically significant relationship with mortality (Fig. 4, **Supplementary Figs. 3,4**).

In patients with CKM stages 3–4, the predictive efficacy of various anthropometric indices for all-cause, cardiovascular, and non-cardiovascular mortality is generally low (**Supplementary Table 9**). Most indices show AUC values ranging from 0.52 to 0.55, and the majority are not statistically significant. This suggests that as CKM progresses to the middle and late stages, the predictive power of anthropometric indices for mortality declines (Fig. 4, **Supplementary Figs. 3,4**).

### 3.6 Discriminative Performance of Anthropometric Indices for Advanced CKM Stage

The discriminative performance of various anthropometric indices for identifying advanced CKM (stage 3/4) is summarised in **Supplementary Table 10**. Among them, ABSI demonstrated the highest discriminative ability (AUC = 0.73), followed by WWI (AUC = 0.70), C-index (AUC = 0.69), and WHR (AUC = 0.64). While most other indices (BMI, WC, BRI, WHtR, VAI, LAP) were significantly associated with higher-stage CKM (*p* < 0.001), BAI and RFM showed limited discriminative value (AUC = 0.51 vs. 0.48, **Supplementary Fig. 5**).

### 3.7 Sensitivity Analysis

After further adjusting for confounding factors (frailty score, antidiabetic medication, antihypertensive medication, lipid-lowering medication), the **Supplementary Table 11** demonstrated the association between obesity-related indicators and mortality in stage 1–2 CKM patients, revealing that Q4 of BMI, WWI, and ABSI were linked to increased all-cause mortality (HR 0.80 vs. 1.18 vs. 1.41, all *p* < 0.05); Q4 of BRI, WWI, ABSI, WHtR, and C-index were linked to increased cardiovascular mortality (HR 1.35 vs. 1.43 vs. 1.60 vs. 1.47 vs. 1.51, all *p* < 0.05); Q4 of BMI, WC, BRI, and ABSI, were linked to increased non-cardiovascular mortality (HR 0.76 vs. 0.85 vs. 0.82 vs. 1.35, all *p* < 0.05). The **Supplementary Table 12** demonstrated the association between obesity-related indicators and mortality in stage 3–4 CKM patients, revealing that Q4 of BMI, WC, BRI, RFM, WHtR and C-index were linked to increased all-cause mortality (HR 0.78 vs. 0.78 vs. 0.76 vs. 0.70 vs. 0.76 vs. 0.87, all *p* < 0.05); Q4 of BRI and ABSI were linked to increased cardiovascular mortality (HR 0.80 vs. 1.37, all *p* < 0.05); Q4 of BMI, WC, WWI, BRI, RFM, WHtR and C-index were linked to increased non-cardiovascular mortality (HR 0.76 vs. 0.77 vs. 0.74 vs. 0.83 vs. 0.68 vs. 0.72 vs. 0.83, all *p* < 0.05).

After excluding patients who died within the 2-year follow-up period, the relationship between obesity-related indices and mortality rates in patients with stage 3–4 CKM is shown in the **Supplementary Table 13**. Q4 of BMI, WC, BRI, and ABSI were linked to increased all-cause mortality (HR 0.91 vs. 0.92 vs. 0.88 vs. 1.28, all *p* < 0.05) and non-cardiovascular mortality (HR 0.83 vs. 0.88 vs. 0.85 vs. 1.26, all *p* < 0.05). Q4 of WWI was linked to increased cardiovascular mortality (HR 1.27, *p* = 0.022). Q2 of C-index was linked to increased all-cause mortality (HR 0.90, *p* = 0.046) and non-cardiovascular mortality (HR 0.84, *p* = 0.003).

## 4. Discussion

Based on a large NHANES cohort (N = 28,911), this study is the first to systematically evaluate the associations between 13 anthropometric indices and both mortality and CKM staging status. Higher quartiles of BMI, WC, WWI, and ABSI were significantly associated with increased risks of all-cause, cardiovascular, and non-cardiovascular mortality among individuals with CKM stages 1–2. In participants with stages 3–4, the predictive utility of most indices declined. However, WWI, C-index, and WHtR remained significantly associated with advanced CKM stage, with ABSI demonstrating the highest discriminatory performance. Importantly, as CKM staging was assessed cross-sectionally, observed associations with advanced stage do not imply causality. These findings support the relevance of anthropometric indices for early risk stratification in CKM syndrome.

Composite anthropometric indices such as BMI, ABSI, and WWI integrate body weight, waist circumference, and height, accounting for their nonlinear relationships. These measures provide a more comprehensive representation of an individual’s overall adiposity burden and body shape composition. BMI is the most commonly used index; however, it cannot distinguish between fat and muscle mass and fails to capture fat distribution. ABSI incorporates body shape factors and has shown superior predictive power for mortality risk and cardio-renal-metabolic diseases compared to traditional BMI [34]. WWI, which combines weight and waist circumference, more sensitively detects central obesity and has gained increasing attention in recent years [35]. Simple anthropometric ratios such as WC, WHR, and WHtR utilize basic body measurements (e.g., waist, hip circumference, height) to reflect the relative distribution of body fat. WHR is used to assess whether fat is predominantly distributed in the abdominal region. WHtR is more suitable for screening visceral fat accumulation [36, 37]. These indicators are easy to obtain and offer practical value for preliminary risk assessment. Shape- and volume-related indices such as BRI, C-index, and AVI can indirectly assess abdominal fat accumulation. BRI reflects the trend of central obesity [38], C-index emphasizes the degree of abdominal fat concentration [39], and AVI estimates abdominal fat volume. These indices help identify fat redistribution and increased intra-abdominal pressure, which may indicate a heightened risk of structural organ damage [40]. Body fat estimation indices such as BAI and RFM are primarily used to estimate total body fat percentage. BAI is calculated from hip circumference and height, while RFM incorporates sex, height, and waist circumference, demonstrating more stable performance in large populations [41, 42]. These measures are suitable for health screening and epidemiological research but are limited in assessing fat distribution and organ-specific damage risk [43]. Visceral fat metabolism-related indices such as LAP and VAI directly reflect visceral fat functionality and are important markers of insulin resistance and lipotoxicity [44]. These indices are of critical importance for the early identification of CKM syndrome risk.

In CKM stages 1–2 (the metabolic risk accumulation phase), our findings are consistent with previous studies: central obesity is a strong driver of mortality risk [45]. Q4 of WC (HR 1.39), WHtR (HR 1.56), and BRI (HR 1.42) were significantly associated with increased cardiovascular mortality risk. Moreover, RCS curves displayed J-shaped patterns (e.g., for WC and WWI), supporting the mechanism by which visceral fat promotes atherosclerosis through inflammation and insulin resistance [46]. ABSI and WWI exhibited broad adverse effects in early CKM stages. The Q4 of ABSI and WWI were significantly associated with increased risks of all-cause mortality (HR 1.43 vs. 1.62), cardiovascular mortality (HR 1.62 vs. 1.38), and non-cardiovascular mortality (HR 1.38 vs. 1.16). These findings align with their focus on height- and weight-adjusted central obesity [47], suggesting that these indices may better capture the pathological effects of visceral fat. In CKM stages 3–4 (the organ damage phase), the associations between obesity indices and mortality shifted: the Q4 of general or abdominal obesity measures such as BMI, WC, and WHtR were associated with decreased risks of all-cause mortality (HRs: 0.87, 0.87, 0.86) and non-cardiovascular mortality (HRs: 0.84, 0.85, 0.81). The RCS curves exhibited a U-shaped pattern, with the lowest mortality risk observed in the overweight range-approximately BMI around 25–27 kg/m^2^ WC around 105 cm, and WHtR around 0.5. This “obesity paradox” has been previously reported in patients with advanced heart failure and CKD [48, 49]. Potential mechanisms include the dominance of muscle wasting (cachexia) as a primary mortality driver in late-stage disease, where moderate fat reserves may serve as an energy buffer [50]. Low BMI may reflect systemic wasting and inflammation, accelerating organ failure. Traditional obesity indices such as BMI cannot distinguish between fat and muscle mass [51]. However, in advanced CKM stages, higher quartiles of ABSI (HR 1.41) and WWI (HR 1.35) were significantly associated with increased cardiovascular mortality risk. This suggests that even in the context of the “obesity paradox”, visceral fat accumulation continues to exacerbate cardiovascular damage through mechanisms such as thrombogenesis and oxidative stress [52, 53]. The lasting link between ABSI/WWI and mortality in advanced CKM stages likely reflects their ability to capture central fat and body shape, indicating visceral fat, metabolic dysfunction, and inflammation-factors still important in later CKM [54]. In contrast, BMI and WC become less reliable as fluid retention, muscle loss, and illness distort body size, weakening their predictive power for mortality in CKM stages 3–4 [28]. From a clinical perspective, it is important to consider whether newer anthropometric indices such as ABSI and WWI offer meaningful advantages over conventional measures like BMI and WC. In contrast, BMI and WC are already routinely assessed in clinical and screening settings and are easily interpretable for patients and clinicians. Therefore, our findings suggest that ABSI and WWI may have the greatest near-term value as adjunct tools to refine risk stratification in individuals who are already considered at elevated CKM risk (e.g., patients with high WC but ‘normal’ BMI), rather than as immediate replacements for traditional anthropometric measures in general screening.

The most clinically relevant finding is the superior discriminatory ability of ABSI (AUC = 0.73) for distinguishing advanced CKM stage (stages 3–4), clearly outperforming traditional indices such as BMI (AUC = 0.64) and WC (AUC = 0.62). Its advantage lies in its unique mathematical construction (ABSI = WC / [BMI^2/3^ × height^1/2^]), which adjusts for overall obesity and more specifically reflects abdominal fat accumulation [55]. The expansion of visceral fat drives the upregulation of inflammatory cytokines and adiponectin dysregulation [10], leading to direct damage to vascular endothelium and glomeruli [56], promotion of insulin resistance, and organ fibrosis-pathophysiological mechanisms [57] well supported by experimental research. ABSI also demonstrates better population generalizability compared to WHtR (AUC = 0.65) and WHR (AUC = 0.64), supporting its use as an effective tool for early identification of high-risk individuals and offering a critical window for intensifying lifestyle or pharmacologic interventions. In contrast, BAI and AVI showed poor performance in predicting both mortality and disease progression. Although VAI and LAP demonstrated some associations in early CKM stages, their utility was limited by missing data and diminished predictive value in later stages. Although some individual effect sizes are modest, they may still be meaningful at the population level given the high prevalence of adverse anthropometric profiles and the chronic nature of CKM-related risk. Even small relative increases in mortality risk can be important in higher-risk subgroups (e.g., individuals in advanced CKM stages), where absolute risk is already elevated [58].

Given the growing global burden of obesity and the CKM syndrome, the findings of this study are of significant clinical and public health relevance. Validated anthropometric indices can help in the early identification of high-risk individuals, enabling timely interventions before the onset of irreversible organ damage. ABSI and WWI, in particular, may serve as valuable tools in clinical risk stratification algorithms due to their strong associations with both mortality and CKM progression. Moreover, the diminished predictive capacity of obesity indices in advanced CKM stages underscores the need to shift focus toward multimorbidity management and integrated care in late-stage disease. Highlighting the need for more nuanced obesity management strategies throughout the entire CKM disease continuum. Furthermore, building on previous medicine studies that have applied clustering and machine learning approaches for risk stratification and management [59, 60, 61], future research may consider integrating anthropometric indices into such frameworks to enhance primary and secondary prevention in CKM syndrome. These results have practical value for early intervention. People with higher waist or body fat measures should receive weight management support, including diet, exercise, and behavior guidance. Those at higher risk need closer monitoring of blood sugar, lipids, and blood pressure for timely treatment adjustments. When body measures exceed risk thresholds, clinicians may refer patients to nutrition, exercise, or health coaching programs to improve adherence. These steps complement existing cardiovascular and kidney risk management guidelines.

### Limitation

Our study has several limitations. First, as an observational study, it is difficult to establish a causal relationship between anthropometric indices and mortality risk in CKM patients. In addition, the discriminatory ability of these indices for advanced CKM stage was assessed using cross-sectional data, which limits inferences about temporal or causal associations. Further prospective studies are needed to validate and strengthen these findings. Second, some advanced indices (e.g., VAI and LAP) require laboratory parameters, and the substantial proportion of missing data for hip circumference reduced the sample size, potentially introducing bias or limiting the robustness of the results. Third, the study population was drawn from the NHANES CKM cohort. Data incompleteness and reliance on self-reported information may have led to misclassification of CKM stages, thereby affecting the accuracy of the findings. Fourth, although multiple potential confounders were adjusted for, residual confounding due to unmeasured variables (e.g., dietary patterns, medication use) cannot be ruled out and may have affected the reliability of the results. Fifth, we excluded participants with missing key covariates or incomplete follow-up. Although this improves model consistency, it may introduce selection bias if excluded individuals differ from those included in health status, access to care, or mortality risk. As a result, the observed associations may not fully reflect the true relationships in the broader population. Lastly, the findings may not be generalizable to populations outside the United States, which may limit the external applicability of the study. Further validation in diverse populations is needed to assess the generalizability of these findings, particularly in non-CKM populations and those with different racial or regional backgrounds. Additional prospective, multicenter studies and replication in external cohorts are necessary to supplement and refine these conclusions.

## 5. Conclusions

This study demonstrated that multiple anthropometric indices, particularly ABSI, WWI, WHtR, and C-index, are significantly associated with both mortality and advanced CKM stage. The findings highlight the potential clinical utility of incorporating such indices into risk stratification frameworks for earlier identification of high-risk individuals. While causal inference remains limited by the observational nature of the study, these results support the need for further prospective research to evaluate whether targeted interventions guided by anthropometric profiles can improve outcomes and inform precision prevention strategies for CKM syndrome.

## Availability of Data and Materials

Data from the National Health and Nutrition Examination Survey (NHANES) are publicly accessible. Interested researchers can obtain the data by making request through the NHANES website at https://www.cdc.gov/nchs/nhanes/index.html.

## Supplementary Material

Supplementary material associated with this article can be found, in the online version, at https://doi.org/10.31083/RCM46650.

## References

[1] Ndumele CE, Rangaswami J, Chow SL, Neeland IJ, Tuttle KR, Khan SS (2023). Cardiovascular-Kidney-Metabolic Health: A Presidential Advisory From the American Heart Association. *Circulation*.

[2] Kidney Disease: Improving Global Outcomes (KDIGO) CKD Work Group (2024). KDIGO 2024 Clinical Practice Guideline for the Evaluation and Management of Chronic Kidney Disease. *Kidney International*.

[3] Zhu R, Wang R, He J, Wang L, Chen H, Wang Y (2025). Associations of cardiovascular-kidney-metabolic syndrome stages with premature mortality and the role of social determinants of health. *The Journal of Nutrition, Health & Aging*.

[4] Sebastian SA, Padda I, Johal G (2024). Cardiovascular-Kidney-Metabolic (CKM) syndrome: A state-of-the-art review. *Current Problems in Cardiology*.

[5] Piché ME, Tchernof A, Després JP (2020). Obesity Phenotypes, Diabetes, and Cardiovascular Diseases. *Circulation Research*.

[6] Shafran I, Krakauer NY, Krakauer JC, Goshen A, Gerber Y (2024). The predictive ability of ABSI compared to BMI for mortality and frailty among older adults. *Frontiers in Nutrition*.

[7] Aoki KC, Mayrovitz HN (2022). Utility of a Body Shape Index Parameter in Predicting Cardiovascular Disease Risks. *Cureus*.

[8] Wang X, Yang S, He G, Xie L (2023). The association between weight-adjusted-waist index and total bone mineral density in adolescents: NHANES 2011-2018. *Frontiers in Endocrinology*.

[9] Du T, Yuan G, Zhang M, Zhou X, Sun X, Yu X (2014). Clinical usefulness of lipid ratios, visceral adiposity indicators, and the triglycerides and glucose index as risk markers of insulin resistance. *Cardiovascular Diabetology*.

[10] Kolb H (2022). Obese visceral fat tissue inflammation: from protective to detrimental?. *BMC Medicine*.

[11] Bae J, Ju JW, Lee S, Nam K, Kim TK, Jeon Y (2022). Association Between Abdominal Fat and Mortality in Patients Undergoing Cardiovascular Surgery. *The Annals of Thoracic Surgery*.

[12] Kruszewska J, Cudnoch-Jedrzejewska A, Czarzasta K (2022). Remodeling and Fibrosis of the Cardiac Muscle in the Course of Obesity-Pathogenesis and Involvement of the Extracellular Matrix. *International Journal of Molecular Sciences*.

[13] Smit M, Werner MJM, Lansink-Hartgring AO, Dieperink W, Zijlstra JG, van Meurs M (2016). How central obesity influences intra-abdominal pressure: a prospective, observational study in cardiothoracic surgical patients. *Annals of Intensive Care*.

[14] D’Onofrio G, Kirschner J, Prather H, Goldman D, Rozanski A (2023). Musculoskeletal exercise: Its role in promoting health and longevity. *Progress in Cardiovascular Diseases*.

[15] Vincent HK, Raiser SN, Vincent KR (2012). The aging musculoskeletal system and obesity-related considerations with exercise. *Ageing Research Reviews*.

[16] Shao X, Yu J, Liu Q, Fu Y, Chen A, Bai G (2025). Systemic inflammation response index mediates the association between relative fat mass and psoriasis risk: a population-based study. *Lipids in Health and Disease*.

[17] Perona JS, Schmidt Rio-Valle J, Ramírez-Vélez R, Correa-Rodríguez M, Fernández-Aparicio Á, González-Jiménez E (2019). Waist circumference and abdominal volume index are the strongest anthropometric discriminators of metabolic syndrome in Spanish adolescents. *European Journal of Clinical Investigation*.

[18] Han F, Guo H, Zhang H, Zheng Y (2025). hs-CRP/HDL-C can predict the risk of all cause mortality in cardiovascular-kidney-metabolic syndrome stage 1-4 patients. *Frontiers in Endocrinology*.

[19] Dong B, Chen Y, Yang X, Chen Z, Zhang H, Gao Y (2025). Estimated glucose disposal rate outperforms other insulin resistance surrogates in predicting incident cardiovascular diseases in cardiovascular-kidney-metabolic syndrome stages 0-3 and the development of a machine learning prediction model: a nationwide prospective cohort study. *Cardiovascular Diabetology*.

[20] Xu X, Li X, Li X, Xue B, Zheng X, Xiao S (2025). Prediction of depression risk in middle-aged and elderly Cardiovascular-Kidney-Metabolic syndrome patients by social and environmental determinants of health: an interpretable machine learning approach using longitudinal data from China. *Journal of Health, Population, and Nutrition*.

[21] Zhu S, Zhang H, Liu Y, Bu W, Wu Q, Wang J (2025). Development of an optimized risk evaluation system for cardiovascular-kidney-metabolic syndrome-associated coronary heart disease based on tabular prior-data fitted network. *Digital Health*.

[22] Quaggin SE, Magod B (2024). A united vision for cardiovascular-kidney-metabolic health. *Nature Reviews. Nephrology*.

[23] Zhang N, Liu X, Wang L, Zhang Y, Xiang Y, Cai J (2024). Lifestyle factors and their relative contributions to longitudinal progression of cardio-renal-metabolic multimorbidity: a prospective cohort study. *Cardiovascular Diabetology*.

[24] Bhaskaran K, Dos-Santos-Silva I, Leon DA, Douglas IJ, Smeeth L (2018). Association of BMI with overall and cause-specific mortality: a population-based cohort study of 3•6 million adults in the UK. *The Lancet. Diabetes & Endocrinology*.

[25] Lin H, Jia X, Yin Y, Li M, Zheng R, Xu Y (2025). Association of body roundness index with cardiovascular disease and all-cause mortality among Chinese adults. *Diabetes, Obesity & Metabolism*.

[26] Lv Y, Zhang Y, Li X, Gao X, Ren Y, Deng L (2024). Body mass index, waist circumference, and mortality in subjects older than 80 years: a Mendelian randomization study. *European Heart Journal*.

[27] Zhu Y, Zou H, Guo Y, Luo P, Meng X, Li D (2023). Associations between metabolic score for visceral fat and the risk of cardiovascular disease and all-cause mortality among populations with different glucose tolerance statuses. *Diabetes Research and Clinical Practice*.

[28] Zhang C, Hao C, Liang W, Hu K, Guo T, Chen Y (2025). Abdominal obesity and frailty progression in population across different Cardiovascular-Kidney-Metabolic syndrome stages: a nationwide longitudinal study. *Diabetology & metabolic syndrome*.

[29] Chen Y, Wu S, Liu H, Zhong Z, Bucci T, Wang Y (2025). Role of oxidative balance score in staging and mortality risk of cardiovascular-kidney-metabolic syndrome: Insights from traditional and machine learning approaches. *Redox Biology*.

[30] von Elm E, Altman DG, Egger M, Pocock SJ, Gøtzsche PC, Vandenbroucke JP (2007). The Strengthening the Reporting of Observational Studies in Epidemiology (STROBE) statement: guidelines for reporting observational studies. *Annals of Internal Medicine*.

[31] Wu S, Zhu J, Lyu S, Wang J, Shao X, Zhang H (2025). Impact of DNA-Methylation Age Acceleration on Long-Term Mortality Among US Adults With Cardiovascular-Kidney-Metabolic Syndrome. *Journal of the American Heart Association*.

[32] Khan SS, Coresh J, Pencina MJ, Ndumele CE, Rangaswami J, Chow SL (2023). Novel Prediction Equations for Absolute Risk Assessment of Total Cardiovascular Disease Incorporating Cardiovascular-Kidney-Metabolic Health: A Scientific Statement From the American Heart Association. *Circulation*.

[33] Levey AS, de Jong PE, Coresh J, El Nahas M, Astor BC, Matsushita K (2011). The definition, classification, and prognosis of chronic kidney disease: a KDIGO Controversies Conference report. *Kidney International*.

[34] Park Y, Kim NH, Kwon TY, Kim SG (2018). A novel adiposity index as an integrated predictor of cardiometabolic disease morbidity and mortality. *Scientific Reports*.

[35] Wen Z, Li X (2023). Association between weight-adjusted-waist index and female infertility: a population-based study. *Frontiers in Endocrinology*.

[36] Zhang FL, Ren JX, Zhang P, Jin H, Qu Y, Yu Y (2021). Strong Association of Waist Circumference (WC), Body Mass Index (BMI), Waist-to-Height Ratio (WHtR), and Waist-to-Hip Ratio (WHR) with Diabetes: A Population-Based Cross-Sectional Study in Jilin Province, China. *Journal of Diabetes Research*.

[37] Rico-Martín S, Calderón-García JF, Sánchez-Rey P, Franco-Antonio C, Martínez Alvarez M, Sánchez Muñoz-Torrero JF (2020). Effectiveness of body roundness index in predicting metabolic syndrome: A systematic review and meta-analysis. *Obesity Reviews*.

[38] Hafezi SG, Saberi-Karimian M, Ghasemi M, Ghamsary M, Moohebati M, Esmaily H (2024). Prediction of the 10-year incidence of type 2 diabetes mellitus based on advanced anthropometric indices using machine learning methods in the Iranian population. *Diabetes Research and Clinical Practice*.

[39] Feng X, Zhu J, Hua Z, Yao S, Tong H (2024). Comparison of obesity indicators for predicting cardiovascular risk factors and multimorbidity among the Chinese population based on ROC analysis. *Scientific Reports*.

[40] Mansoori A, Allahyari M, Mirvahabi MS, Tanbakuchi D, Ghoflchi S, Derakhshan-Nezhad E (2024). Predictive properties of novel anthropometric and biochemical indexes for prediction of cardiovascular risk. *Diabetology & Metabolic Syndrome*.

[41] Lokpo SY, Ametefe CY, Osei-Yeboah J, Owiredu WKBA, Ahenkorah-Fondjo L, Agordoh PD (2023). Performance of Body Adiposity Index and Relative Fat Mass in Predicting Bioelectric Impedance Analysis-Derived Body Fat Percentage: A Cross-Sectional Study among Patients with Type 2 Diabetes in the Ho Municipality, Ghana. *BioMed Research International*.

[42] Encarnação IGA, Cerqueira MS, Silva DAS, Marins JCB, Magalhães PM (2022). Prediction of body fat in adolescents: validity of the methods relative fat mass, body adiposity index and body fat index. *Eating and Weight Disorders: EWD*.

[43] Zhu X, Yue Y, Li L, Zhu L, Cai Y, Shu Y (2024). The relationship between depression and relative fat mass (RFM): A population-based study. *Journal of Affective Disorders*.

[44] Huang Y, Zhao D, Yang Z, Wei C, Qiu X (2025). The relationship between VAI, LAP, and depression and the mediation role of sleep duration-evidence from NHANES 2005-2020. *BMC Psychiatry*.

[45] Sahakyan KR, Somers VK, Rodriguez-Escudero JP, Hodge DO, Carter RE, Sochor O (2015). Normal-Weight Central Obesity: Implications for Total and Cardiovascular Mortality. *Annals of Internal Medicine*.

[46] Vgontzas AN, Bixler EO, Chrousos GP (2003). Metabolic disturbances in obesity versus sleep apnoea: the importance of visceral obesity and insulin resistance. *Journal of Internal Medicine*.

[47] Chen ZT, Wang XM, Zhong YS, Zhong WF, Song WQ, Wu XB (2024). Association of changes in waist circumference, waist-to-height ratio and weight-adjusted-waist index with multimorbidity among older Chinese adults: results from the Chinese longitudinal healthy longevity survey (CLHLS). *BMC Public Health*.

[48] Alebna PL, Mehta A, Yehya A, daSilva-deAbreu A, Lavie CJ, Carbone S (2024). Update on obesity, the obesity paradox, and obesity management in heart failure. *Progress in Cardiovascular Diseases*.

[49] Alzayer H, Roshanravan B (2023). Dissecting the Obesity Paradox in Patients With Obesity and CKD. *Kidney International Reports*.

[50] Bielecka-Dabrowa A, Ebner N, Dos Santos MR, Ishida J, Hasenfuss G, von Haehling S (2020). Cachexia, muscle wasting, and frailty in cardiovascular disease. *European Journal of Heart Failure*.

[51] Suthahar N, Zwartkruis V, Geelhoed B, Withaar C, Meems LMG, Bakker SJL (2024). Associations of relative fat mass and BMI with all-cause mortality: Confounding effect of muscle mass. *Obesity*.

[52] Tutor AW, Lavie CJ, Kachur S, Milani RV, Ventura HO (2023). Updates on obesity and the obesity paradox in cardiovascular diseases. *Progress in Cardiovascular Diseases*.

[53] McMurray F, Patten DA, Harper ME (2016). Reactive Oxygen Species and Oxidative Stress in Obesity-Recent Findings and Empirical Approaches. *Obesity*.

[54] Bihari M, Habánová M, Jančichová K, Gažarová M (2022). Diagnosis of obesity and evaluation of the risk of premature death (ABSI) based on body mass index and visceral fat area. *Roczniki Panstwowego Zakladu Higieny*.

[55] Calderón-García JF, Roncero-Martín R, Rico-Martín S, De Nicolás-Jiménez JM, López-Espuela F, Santano-Mogena E (2021). Effectiveness of Body Roundness Index (BRI) and a Body Shape Index (ABSI) in Predicting Hypertension: A Systematic Review and Meta-Analysis of Observational Studies. *International Journal of Environmental Research and Public Health*.

[56] Sabaratnam R, Svenningsen P (2021). Adipocyte-Endothelium Crosstalk in Obesity. *Frontiers in Endocrinology*.

[57] Pellegrinelli V, Rodriguez-Cuenca S, Rouault C, Figueroa-Juarez E, Schilbert H, Virtue S (2022). Dysregulation of macrophage PEPD in obesity determines adipose tissue fibro-inflammation and insulin resistance. *Nature Metabolism*.

[58] Ostrominski JW, Harrington J, Claggett BL, Filippatos G, Desai AS, Jhund PS (2025). Anthropometric Measures, Cardiovascular Outcomes, and Treatment Effects of Finerenone in Cardiovascular-Kidney-Metabolic Disease: Pooled Participant-Level Analysis of 3 Global Trials. *Journal of the American College of Cardiology*.

[59] Chen Y, Gue Y, Banach M, Mikhailidis D, Toth PP, Gierlotka M (2024). Phenotypes of Polish primary care patients using hierarchical clustering: Exploring the risk of mortality in the LIPIDOGEN2015 study cohort. *European Journal of Clinical Investigation*.

[60] Chen Y, Huang B, Calvert P, Liu Y, Gue Y, Gupta D (2024). Phenotypes of South Asian patients with atrial fibrillation and holistic integrated care management: cluster analysis of data from KERALA-AF Registry. *The Lancet Regional Health. Southeast Asia*.

[61] Yang Z, Li Y, Liu Y, Zhong Z, Ditchfield C, Guo T (2024). Prognostic effects of glycaemic variability on diastolic heart failure and type 2 diabetes mellitus: insights and 1-year mortality machine learning prediction model. *Diabetology & Metabolic Syndrome*.

