# Supplementary material for: The Associations of Anthropometric Indices With Stages and Mortality in Cardiovascular–Kidney–Metabolic Syndrome: Insights From NHANES
Source: Rev Cardiovasc Med. 2026 Feb 25;27(2):46650. doi: 10.31083/RCM46650 (PMC12960012; doi:10.31083/RCM46650)
Supplement: Supplementary file 1 [file 2153-8174-27-2-46650-s1.zip › Supplementary Material.docx]

Supplementary Table 1. Checklist for Strengthening the Reporting of Observational Studies in Epidemiology (STROBE) for this cohort study

Supplementary Table 2. List of 40 variables included in the score of Frailty score

Supplementary Table 3. Definitions of CKM

Supplementary Table 4. Detailed algorithm for evaluating each CKM stage

Supplementary Table 5. Detailed algorithm of the simplified 10-year cardiovascular disease risk models

Supplementary Table 6. Assessment of Obesity-Related Indices

Supplementary Table 7. The interaction between obesity-related indices and CKM progression

Supplementary Table 8. Predictive efficacy of obesity-related indices for mortality outcomes in CKM stages 1-2 patients

Supplementary Table 9. Predictive efficacy of obesity-related indices for mortality outcomes in CKM stages 3-4 patients

Supplementary Table 10. Predictive efficacy of obesity-related indices for CKM progression in all CKM patients

Supplementary Table 11. Sensitivity analysis of obesity-related indices and mortality outcomes in CKM stage 1-2 patients after adjusting other confounding factors

Supplementary Table 12. Sensitivity analysis of obesity-related indices and mortality outcomes in CKM stage 3-4 patients after adjusting other confounding factors

Supplementary Table 13. Sensitivity analysis of obesity-related indices and mortality outcomes in CKM stage 3-4 patients after excluding patients who died within two years

Supplementary Fig. 1. Flowchart of this study.

Supplementary Fig. 2. Mortality in different CKM stages.

Supplementary Fig. 3. Receiver Operating Characteristic Crues for Associations Between Obesity-related indices and Mortality Outcomes in CKM stages 1-2 and 3-4 Patients.

Supplementary Fig. 4. Receiver Operating Characteristic Crues for Associations Between Obesity-related indices and Mortality Outcomes in CKM stage 1-2 and 3-4 Patients.

Supplementary Fig. 5. Receiver Operating Characteristic Crues for Associations Between Obesity-related indices and CKM progression.

Supplementary Table 1. Checklist for Strengthening the Reporting of Observational Studies in Epidemiology (STROBE) for this cohort study

|  | Item No | Recommendation | Page No |
| --- | --- | --- | --- |
| **Title and abstract** | 1 | (*a*) Indicate the study’s design with a commonly used term in the title or the abstract | 1 |
|  |  | (*b*) Provide in the abstract an informative and balanced summary of what was done and what was found | 1 |
| Introduction | | | |
| Background/rationale | 2 | Explain the scientific background and rationale for the investigation being reported | 2 |
| Objectives | 3 | State specific objectives, including any prespecified hypotheses | 2 |
| Methods | | | |
| Study design | 4 | Present key elements of study design early in the paper | 3 |
| Setting | 5 | Describe the setting, locations, and relevant dates, including periods of recruitment, exposure, follow-up, and data collection | 2,3 |
| Participants | 6 | (*a*) Give the eligibility criteria, and the sources and methods of selection of participants. Describe methods of follow-up | 3 |
|  |  | (*b*) For matched studies, give matching criteria and number of exposed and unexposed | N/A |
| Variables | 7 | Clearly define all outcomes, exposures, predictors, potential confounders, and effect modifiers. Give diagnostic criteria, if applicable | 3 |
| Data sources/ measurement | 8* | For each variable of interest, give sources of data and details of methods of assessment (measurement). Describe comparability of assessment methods if there is more than one group | 3 |
| Bias | 9 | Describe any efforts to address potential sources of bias | 3,6 |
| Study size | 10 | Explain how the study size was arrived at | N/A |
| Quantitative variables | 11 | Explain how quantitative variables were handled in the analyses. If applicable, describe which groupings were chosen and why | 3 |
| Statistical methods | 12 | (*a*) Describe all statistical methods, including those used to control for confounding | 3,6 |
|  |  | (*b*) Describe any methods used to examine subgroups and interactions | 3 |
|  |  | (*c*) Explain how missing data were addressed | 3 |
|  |  | (*d*) If applicable, explain how loss to follow-up was addressed | 3 |
|  |  | (*e*) Describe any sensitivity analyses | 6 |
| Results | | |  |
| Participants | 13* | (a) Report numbers of individuals at each stage of study—eg numbers potentially eligible, examined for eligibility, confirmed eligible, included in the study, completing follow-up, and analysed | 3 |
|  |  | (b) Give reasons for non-participation at each stage | 3 |
|  |  | (c) Consider use of a flow diagram | 3 |
| Descriptive data | 14* | (a) Give characteristics of study participants (eg demographic, clinical, social) and information on exposures and potential confounders | 6 |
|  |  | (b) Indicate number of participants with missing data for each variable of interest | 3 |
|  |  | (c) Summarise follow-up time (eg, average and total amount) | 3 |
| Outcome data | 15* | Report numbers of outcome events or summary measures over time | 6 |
| Main results | 16 | (*a*) Give unadjusted estimates and, if applicable, confounder-adjusted estimates and their precision (eg, 95% confidence interval). Make clear which confounders were adjusted for and why they were included | 6-11 |
|  |  | (*b*) Report category boundaries when continuous variables were categorized | 6-11 |
|  |  | (*c*) If relevant, consider translating estimates of relative risk into absolute risk for a meaningful time period | 6-11 |
| Other analyses | 17 | Report other analyses done-eg analyses of subgroups and interactions, and sensitivity analyses | 11-12 |
| Discussion | | | |
| Key results | 18 | Summarise key results with reference to study objectives | 12 |
| Limitations | 19 | Discuss limitations of the study, taking into account sources of potential bias or imprecision. Discuss both direction and magnitude of any potential bias | 14 |
| Interpretation | 20 | Give a cautious overall interpretation of results considering objectives, limitations, multiplicity of analyses, results from similar studies, and other relevant evidence | 12-14 |
| Generalisability | 21 | Discuss the generalisability (external validity) of the study results | 12-14 |
| Other information | | | |
| Funding | 22 | Give the source of funding and the role of the funders for the present study and, if applicable, for the original study on which the present article is based | 15 |

*Give information separately for exposed and unexposed groups

**Note:** An Explanation and Elaboration article discusses each checklist item and gives methodological background and published examples of transparent reporting. The STROBE checklist is best used in conjunction with this article (freely available on the Web sites of PLoS Medicine at http://www.plosmedicine.org/, Annals of Internal Medicine at http://www.annals.org/, and Epidemiology at http://www.epidem.com/). Information on the STROBE Initiative is available at http://www.strobe-statement.org

Supplementary Table 2. List of 40 variables included in the score of Frailty score

| **Variables** | **Cut point** |
| --- | --- |
| Comorbidities | ﻿Yes = 1, Suspect = 0.5, No = 0 |
| High blood pressure | ﻿Yes = 1, Suspect = 0.5, No = 0 |
| Heart attack | ﻿Yes = 1, Suspect = 0.5, No = 0 |
| Chronic heart failure | ﻿Yes = 1, Suspect = 0.5, No = 0 |
| Stroke | ﻿Yes = 1, Suspect = 0.5, No = 0 |
| Cancer | ﻿Yes = 1, Suspect = 0.5, No = 0 |
| Diabetes | ﻿Yes = 1, Suspect = 0.5, No = 0 |
| Arthritis | ﻿Yes = 1, Suspect = 0.5, No = 0 |
| Chronic lung disease | ﻿Yes = 1, Suspect = 0.5, No = 0 |
| Dependence |  |
| Help bathing | ﻿Yes = 1, No = 0 |
| Help dressing | ﻿Yes = 1, No = 0 |
| Help getting in/out of chair | ﻿Yes = 1, No = 0 |
| Help walking around house | ﻿Yes = 1, No = 0 |
| Help eating | ﻿Yes = 1, No = 0 |
| Help grooming | ﻿Yes = 1, No = 0 |
| Help using toilet | ﻿Yes = 1, No = 0 |
| Help up/down stairs | ﻿Yes = 1, No = 0 |
| Help lifting 10 lbs | ﻿Yes = 1, No = 0 |
| Help shopping | ﻿Yes = 1, No = 0 |
| Help with housework | ﻿Yes = 1, No = 0 |
| Help with meal preparations | ﻿Yes = 1, No = 0 |
| Help taking medication | ﻿Yes = 1, No = 0 |
| Help with finances | ﻿Yes = 1, No = 0 |
| Depression |  |
| Feel everything is an effort | ﻿Most of time = 1, Some time = 0.5, Rarely = 0 |
| Feel depressed | ﻿Most of time = 1, Some time = 0.5, Rarely = 0 |
| Feel happy | ﻿Most of time = 1, Some time = 0.5, Rarely = 0 |
| Feel lonely | ﻿Most of time = 1, Some time = 0.5, Rarely = 0 |
| Have trouble getting going | ﻿Most of time = 1, Sometime = 0.5, Rarely = 0 |
| MMSE | ﻿<10 = 1, 11–17 = 0.75, 18–20 = 0.5, 20–24 = 0.25, >24 = 0 |
| General health |  |
| Lost more than 10 lbs in last year | ﻿Yes = 1, No = 0 |
| Self-rating of health | ﻿Poor = 1, Fair = 0.75, Good = 0.5, V. Good = 0.25, Excellent = 0 |
| How health has changed in last year | ﻿Worse = 1, Better/Same = 0 |
| Stayed in bed at least half the day due to health (in last month) | ﻿Yes = 1, No = 0 |
| Cut down on usual activity (in last month) | ﻿Yes = 1, No = 0 |
| Walk outside | ﻿ <3 days = 1, ≤ 3 days = 0 |
| Physical performance and anthropometry |  |
| Peak flow (liters/min) | ﻿≤ 340 for men, ﻿≤ 310 for women |
| Shoulder strength (kg) | ﻿≤ 12 for men, ﻿≤ 9 for women |
| Body mass index (kg/m^2^) | ﻿<18.5, ≥ 30 as a deficit, 25-<30 as a 'half deficit' |
| Grip strength (kg) | ﻿In men, for BMI ≤ 24, GS ≤ 29, for BMI 24.1–28, GS ≤ 30, for BMI >28, GS ≤ 32  In women, ﻿for BMI ≤ 23, GS ≤ 17, for BMI 23.1–26, GS ≤ 17.3, for BMI 26.1–29, GS ≤ 18, for BMI>29, GS ≤ 21 |
| Usual pace (sec) | ﻿>16 |
| Rapid pace (sec) | ﻿>10 |

Frailty score: validated composite frailty index based on previously published method. Higher values indicate greater frailty.

Abbreviations: BMI = body mass index; GS = grip strength; MMSE = Mini-Mental State Examination.

Supplementary Table 3. Definitions of CKM

| **CKM conditions** | **Definition** | **CKM indicators** | **Threshold for CKM indicators** |
| --- | --- | --- | --- |
| CVD | Individuals with clinical CVD or subclinical CVD | Clinical CVD | History of chronic heart failure, coronary heart disease, heart attack, or stroke |
|  |  | Subclinical CVD | Any of the following criterion is met:  1) Very high-risk CKD in KDIGO classification: UACR ≥ 300 mg/g and eGFR ≤ 45-59 ml/min/1.73m2, UACR ≥ 30 mg/g and eGFR ≤ 30-44 ml/min/1.73m2, or eGFR ≤ 29 ml/min/1.73m2.  2) Predicted 10-year CVD risk ≥ 20% |
| Kidney diseases | Individuals with CKD | CKD | Moderate-to-high-risk CKD in KDIGO classification: UACR ≥ 30 mg/g and eGFR ≥ 60 ml/min/1.73m2, UACR < 300 mg/g and eGFR ≤ 45-59 ml/min/1.73m2, or UACR < 30 mg/g and eGFR ≤ 30-44 ml/min/1.73m2. |
| Metabolic disorders | Individuals with overweight/obesity, abdominal obesity, prediabetes, diabetes, hypertension, hypertriglyceridemia or MetS | Overweight/obesity | BMI ≥25 kg/m2 (or ≥23 kg/m2 if Asian ancestry) * |
|  |  | Abdominal obesity | Waist circumference ≥88/102 cm in female/male (or if Asian ancestry ≥80/90 cm in female/male) |
|  |  | Prediabetes | Fasting blood glucose ≥ 100-124 mg/dL or HbA1c ≥ 5.7%-6.4% and without self-reported diagnosis of diabetes, use of insulin, or oral hypoglycemic agents |
|  |  | Diabetes | Fasting blood glucose ≥ 125 mg/dL or HbA1c ≥ 6.5% or self-reported diagnosis of diabetes, use of insulin, or oral hypoglycemic agents |
|  |  | Hypertension | SBP ≥130 mm Hg or DBP ≥80 mm Hg or self-reported diagnosis of hypertension or use of antihypertensive medications |
|  |  | Hypertriglyceridemia | Triglycerides ≥ 135 mg/dL |
|  |  | MetS | MetS is defined by the presence of 3 or more of the following:   \| 1) Waist circumference ≥88/102 cm in female/male (or if Asian ancestry ≥80/90 cm in female/male).  2) HDL cholesterol <50/40 mg/dL in female/male.  3) Triglycerides ≥150 mg/dL.  4) Elevated blood pressure (SBP ≥130 mm Hg or DBP ≥80 mm Hg and/or use of antihypertensive medications)  5) Fasting blood glucose ≥100 mg/dL \| \| --- \| |

*Asian was not listed as a separate race/ethnicity until NAHNES 2011-2012, therefore the uniform threshold for BMI and waist circumference was used in all participants in NHANES 1999-2010.

Abbreviations: BMI = body mass index; CKD = chronic kidney disease; CKM = cardiovascular-kidney-metabolic syndrom; CVD = cardiovascular disease; DBP = diastolic blood pressure; eGFR = estimated glomerular filtration rate; HDL-C = high-density lipoprotein cholesterol; KDIGO = The Kidney Disease: Improving Global Outcomes; MetS = metabolic syndrome; SBP = systolic blood pressure; UACR = urinary albumin to creatinine ratio.

Supplementary Table 4. Detailed algorithm for evaluating each CKM stage

| **CKM stages** | **Definition** | **Criterion** | **Threshold for CKM conditions** |
| --- | --- | --- | --- |
| Stage 0: No CKM risk factors | Individuals with normal BMI and waist circumference, normoglycemia, normotension, a normal lipid profile, and no evidence of CKD or subclinical or clinical CVD | All criteria are met | BMI <25 kg/m2 (or <23 kg/m2 if Asian ancestry)* |
|  |  |  | Waist circumference <88/102 cm in female/male (or if Asian ancestry <80/90 cm in female/male) |
|  |  |  | Fasting blood glucose < 100 mg/dL and HbA1c < 5.7% and without self-reported diagnosis of diabetes, use of insulin, or oral hypoglycemic agents |
|  |  |  | SBP <130 mm Hg and DBP <80 mm Hg without self-reported diagnosis of hypertension or use of antihypertensive medications |
|  |  |  | HDL cholesterol >50/40 mg/dL in female/male and triglycerides < 150 mg/dL |
|  |  |  | Low-risk CKD in KDIGO classification according to eGFR and UACR: UACR < 30 mg/g and eGFR ≥ 60 ml/min/1.73m2. |
|  |  |  | Predicted 10-year CVD risk < 20% |
|  |  |  | No clinical CVD |
| Stage 1: Excess or dysfunctional adiposity | Individuals with overweight/obesity, abdominal obesity, or dysfunctional adipose tissue, without the presence of other metabolic risk factors or CKD | Any of the three criteria is met | Overweight/obesity |
|  |  |  | Abdominal obesity |
|  |  |  | Prediabetes |
|  |  | All criteria are met | SBP <130 mm Hg and DBP <80 mm Hg without self-reported diagnosis of hypertension or use of antihypertensive medications |
|  |  |  | HDL cholesterol >50/40 mg/dL in female/male and triglycerides <150 mg/dL |
|  |  |  | Low-risk CKD in KDIGO classification according to eGFR and UACR: UACR < 30 mg/g and eGFR ≥ 60 ml/min/1.73m2 |
|  |  |  | Predicted 10-year CVD risk < 20% |
|  |  |  | No clinical CVD |
| Stage 2: Metabolic risk factors and CKD | Individuals with metabolic risk factors (hypertriglyceridemia, hypertension, MetS, diabetes), or CKD | Any of the five criteria is met | Hypertriglyceridemia |
|  |  |  | Hypertension |
|  |  |  | diabetes |
|  |  |  | MetS |
|  |  |  | Moderate-to-high-risk CKD in KDIGO classification |
|  |  | All criteria are met | No very high-risk CKD in KDIGO classification |
|  |  |  | Predicted 10-year CVD risk < 20% |
|  |  |  | No clinical CVD |
| Stage 3: Subclinical CVD in CKM | Subclinical CVD among individuals with excess/dysfunctional adiposity, other metabolic risk factors, or CKD | Any of the two criteria is met | Very high-risk CKD in KDIGO classification |
|  |  |  | Predicted 10-year CVD risk ≥ 20% |
|  |  | Any of the eight criteria is met | Overweight/obesity |
|  |  |  | Abdominal obesity |
|  |  |  | Prediabetes |
|  |  |  | Hypertriglyceridemia |
|  |  |  | Hypertension |
|  |  |  | diabetes |
|  |  |  | MetS |
|  |  |  | Moderate-to-high-risk CKD in KDIGO classification |
|  |  | The criterion is met | No clinical CVD |
| Stage 4: Clinical CVD in CKM | Clinical CVD among individuals with excess/dysfunctional adiposity, other metabolic risk factors, or CKD | The criterion is met | Clinical CVD |
|  |  | Any of the nine criteria is met | Overweight/obesity |
|  |  |  | Abdominal obesity |
|  |  |  | Prediabetes |
|  |  |  | Hypertriglyceridemia |
|  |  |  | Hypertension |
|  |  |  | diabetes |
|  |  |  | MetS |
|  |  |  | Moderate-to-high-risk CKD in KDIGO classification |
|  |  |  | Very high-risk CKD in KDIGO classification |

*Asian was not listed as a separate race/ethnicity until NHANES 2011-2012, therefore the uniform threshold for BMI and waist circumference was used in all participants in NHANES 1999-2010.

Abbreviations: BMI = body mass index; CKD = chronic kidney disease; CKM = cardiovascular-kidney-metabolic syndrom; CVD = cardiovascular disease; DBP = diastolic blood pressure; eGFR = estimated glomerular filtration rate; HDL-C = high-density lipoprotein; KDIGO = The Kidney Disease: Improving Global Outcomes; NHANES = National Health and Nutrition Examination Survey; SBP = systolic blood pressure; UACR = urinary albumin to creatinine ratio.

Supplementary Table 5. Detailed algorithm of the simplified 10-year cardiovascular disease risk models

| **Sex** | **Calculation** |
| --- | --- |
| Women | log-Odds = -3.307728 + 0.7939329 × (age – 55) /10 + 0.0305239 × (TC – HDL-C – 3.5) – 0.1606857 × (HDL-C – 1.3) /0.3 – 0.2394003 × (min(SBP, 110) – 110) /20 + 0.360078 × (max(SBP, 110) – 130) /20 + 0.8667604 × (if diabetes) + 0.5360739 × (if current smoker) + 0.6045917 × (min(eGFR, 60) – 60) / -15 + 0.0433769 × (max(eGFR, 60) – 90) / -15 + 0.3151672 × (if using anti-hypertensive medication) – 0.1477655 × (if using statin) – 0.0663612 × (if using anti-hypertensive medication) × (max(SBP, 110) – 130) /20 + 0.1197879 × (if using statin) × (TC – HDL-C – 3.5) – 0.0819715 × (age – 55) /10 × (TC – HDL-C – 3.5) + 0.0306769 × (age – 55) /10 × (HDL-C – 1.3) /0.3 – 0.0946348 × (age – 55) /10 × (max(SBP, 110) – 130) /20 – 0.27057 × (age – 55) /10 × (if diabetes) – 0.078715 × (age – 55) /10 × (if current smoker) – 0.1637806 × (age – 55) /10 × (min(eGFR, 60) – 60) / -15  Risk = exp(log-Odds) / (1 + exp(log-Odds)) |
| Men | log-Odds = -3.031168 + 0.7688528 × (age – 55) /10 + 0.0736174 × (TC – HDL-C – 3.5) – 0.0954431 × (HDL-C – 1.3) /0.3 – 0.4347345 × (min(SBP, 110) – 110) /20 + 0.3362658 × (max(SBP, 110) – 130) /20 + 0.7692857 × (if diabetes) + 0.4386871 × (if current smoker) + 0.5378979 × (min(eGFR, 60) – 60) / -15 + 0.0164827 × (max(eGFR, 60) – 90) / -15 + 0.288879 × (if using anti-hypertensive medication) – 0.1337349 × (if using statin) – 0.0475924 × (if using anti-hypertensive medication) × (max(SBP, 110) – 130) /20 + 0.150273 × (if using statin) × (TC – HDL-C – 3.5) – 0.0517874 × (age – 55) /10 × (TC – HDL-C – 3.5) + 0.0191169 × (age – 55) /10 × (HDL-C – 1.3) /0.3 – 0.1049477 × (age – 55) /10 × (max(SBP, 110) – 130) /20 – 0.2251948 × (age – 55) /10 × (if diabetes) – 0.0895067 × (age – 55) /10 × (if current smoker) – 0.1543702 × (age – 55) /10 × (min(eGFR, 60) – 60) / -15  Risk = exp(log-Odds) / (1 + exp(log-Odds)) |

Abbreviations: eGFR = estimated glomerular filtration rate; HDL-C = high-density lipoprotein cholesterol; SBP = systolic blood pressure; TC = total cholesterol.

Supplementary Table 6. Assessment of Obesity-Related Indices

| **Variables** | **Calculation formula** |
| --- | --- |
| BMI | BMI = Weight (kg) / Height² (m) |
| WC | WC = Measured in cm |
| BRI | BRI = √((WC (m) / π) / (1 - (Height (m) / WC (m)))) |
| WWI | WWI = WC (cm)/ √Weight (kg) |
| RFM | RFM = 64 - (20 × Height (m) / WC (m)) for male; 76 - (20 × Height (m) / WC (m)) for female |
| ABSI | ABSI = WC (m) / (BMI^2/3^ × Height (m)^1/2^) |
| WHtR | WHtR = WC (cm) / Height (cm) |
| C-index | C-index = WC (m) / 0.109 × √(Weight (kg) / Height (m)) |
| VAI | VAI = WC (cm) /［39.68 + 1.88×BMI (kg/m^2^)］×［TG (mmol/L) /1.03］×［1.31 / HDL-C (mmol/L)］for male; WC (cm) /［36.58+1.89×BMI (kg/m^2^)］×［TG (mmol/L) /0.81］×［1.52 / HDL-C (mmol/L)］for female |
| LAP | LAP = (WC (cm) - 65) × TG (mmol/L) for male; (WC (cm) - 58) × TG (mmol/L) for women |
| WHR | WHR = WC (cm) / Hip circumference (cm) |
| BAI | BAI = Hip circumference (m) / Height (m)^1.5^ - 18 |
| AVI | AVI = 2 × WC (cm)^2^ + 0.7 × (WC (cm) - Hip circumference (cm))^2^ / 1000 |

Abbreviations: ABSI, A Body Shape Index; AVI, Abdominal Volume Index; BMI, Body mass index; BRI, Body Roundness Index; BAI, Body Adiposity Index; C-index, Conicity Index; LAP, Lipid Accumulation Product; RFM, Relative Fat Mass; VAI, Visceral Adiposity Index; WHR, Waist-to-Hip Ratio; WC, waist circumference; WWI, Weight-adjusted Waist Index; WHtR, Waist-to-Height Ratio; TG, triglycerides; HDL-C, high-density lipoprotein cholesterol.

Supplementary Table 7. The interaction between obesity-related indices and CKM progression

|  | **CKM stage 1-2 → CKM stage 3-4** | | | | |
| --- | --- | --- | --- | --- | --- |
|  | **OR (95% CI)** | ***P*** |  | **OR (95% CI)** | ***P*** |
| **BMI** |  |  | **WC** |  |  |
| Q1 | *Reference* |  | Q1 | *Reference* |  |
| Q2 | 1.02 (0.85, 1.22) | 0.830 | Q2 | 1.07 (0.88, 1.29) | 0.501 |
| Q3 | 1.12 (0.93, 1.33) | 0.228 | Q3 | 1.28 (1.06, 1.55) | 0.010 |
| Q4 | 1.19 (1.05, 1.45) | 0.042 | Q4 | 1.98 (1.06, 1.58) | 0.012 |
| **BRI** |  |  | **WWI** |  |  |
| Q1 | *Reference* |  | Q1 | *Reference* |  |
| Q2 | 1.06 (0.88, 1.28) | 0.529 | Q2 | 1.16 (0.91, 1.49) | 0.230 |
| Q3 | 1.30 (1.07, 1.57) | 0.008 | Q3 | 1.12 (0.88, 1.42) | 0.330 |
| Q4 | 1.20 (0.98, 1.48) | 0.073 | Q4 | 1.34 (1.06, 1.71) | 0.014 |
| **ABSI** |  |  | **RFM** |  |  |
| Q1 | *Reference* |  | Q1 | *Reference* |  |
| Q2 | 0.86 (0.66, 1.12) | 0.244 | Q2 | 1.12 (0.92, 1.34) | 0.253 |
| Q3 | 0.99 (0.78, 1.25) | 0.942 | Q3 | 1.28 (0.98, 1.67) | 0.070 |
| Q4 | 1.02 (0.80, 1.28) | 0.882 | Q4 | 1.54 (0.12, 2.12) | 0.007 |
| **WHtR** |  |  | **C-index** |  |  |
| Q1 | *Reference* |  | Q1 | *Reference* |  |
| Q2 | 1.00 (0.82, 1.23) | 0.967 | Q2 | 1.08 (0.86, 1.37) | 0.477 |
| Q3 | 1.05 (0.86, 1.27) | 0.615 | Q3 | 1.07 (0.86, 1.34) | 0.508 |
| Q4 | 1.35 (1.10, 1.65) | 0.004 | Q4 | 1.30 (1.04, 1.61) | 0.020 |

Model: adjusted age, sex, race and ethnicity, poverty income ratio, marital states, education, smoking status, alcohol consumption, physical activity, cardiovascular disease, hypertensive, diabetes, chronic kidney disease, stroke.

Abbreviations: OR, odds ratio; CI, confidence interval; CKM, cardiovascular-kidney-metabolic syndrome; ABSI, A Body Shape Index; BMI, Body mass index; BRI, Body Roundness Index; C-index, Conicity Index; RFM, Relative Fat Mass; WC, waist circumference; WWI, Weight-adjusted Waist Index; WHtR, Waist-to-Height Ratio.

Supplementary Table 8. Predictive efficacy of obesity-related indices for mortality outcomes in CKM stages 1-2 patients

|  | **All-cause death** | | | **Cardiovascular death** | | | | **Non cardiovascular death** | | | |
| --- | --- | --- | --- | --- | --- | --- | --- | --- | --- | --- | --- |
|  | **AUC (95 CI)** | **P** |  | | **AUC (95 CI)** | **P** |  | | **AUC (95 CI)** | **P** |  |
| **ABSI** | 0.65 (0.64, 0.66) | *Reference* |  | | 0.64 (0.61, 0.67) | *Reference* |  | | 0.65 (0.63, 0.66) | *Reference* |  |
| **BMI** | 0.54 (0.53, 0.56) | < 0.001 |  | | 0.50 (0.47, 0.53) | < 0.001 |  | | 0.55 (0.54, 0.57.) | < 0.001 |  |
| **WC** | 0.51 (0.50, 0.52) | < 0.001 |  | | 0.56 (0.53, 0.59) | < 0.001 |  | | 0.49 (0.48, 0.51) | < 0.001 |  |
| **BRI** | 0.50 (0.49, 0.52) | < 0.001 |  | | 0.55 (0.53, 0.58) | < 0.001 |  | | 0.50 (0.48, 0.52) | < 0.001 |  |
| **WWI** | 0.60 (0.59, 0.61) | < 0.001 |  | | 0.61 (0.58, 0.64) | 0.028 |  | | 0.59 (0.58, 0.61) | < 0.001 |  |
| **RFM** | 0.49 (0.48, 0.51) | < 0.001 |  | | 0.51 (0.48, 0.54) | < 0.001 |  | | 0.50 (0.48, 0. 51) | < 0.001 |  |
| **WHtR** | 0.51 (0.50, 0.53) | < 0.001 |  | | 0.55 (0.53, 0.58) | < 0.001 |  | | 0.50 (0.49, 0.52) | < 0.001 |  |
| **C-index** | 0.61 (0.59, 0.62) | < 0.001 |  | | 0.63 (0.60, 0.66) | 0.304 |  | | 0.60(0.58, 0.61) | < 0.001 |  |
| **VAI** | 0.58 (0.56, 0.59) | < 0.001 |  | | 0.57 (0.54, 0.61) | 0.002 |  | | 0.57 (0.55, 0.59) | < 0.001 |  |
| **LAP** | 0.54 (0.52, 0.56) | < 0.001 |  | | 0.57 (0.53, 0.61) | 0.001 |  | | 0.53 (0.51, 0.55) | < 0.001 |  |
| **WHR** | 0.54 (0.43, 0.65) | 0.005 |  | | 0.80 (0.59, 0.98) | 0.949 |  | | 0.50 (0.39, 0.61) | 0.003 |  |
| **BAI** | 0.69 (0.57, 0.81) | 0.541 |  | | 0.76 (0.65, 0.88) | 0.081 |  | | 0.67 (0.53, 0.81) | 0.581 |  |
| **AVI** | 0.62 (0.50, 0.74) | 0.095 |  | | 0.52 (0.25, 0.79) | 0.001 |  | | 0.65 (0.52, 0.78) | 0.326 |  |

Abbreviations: ABSI, A Body Shape Index; AVI, Abdominal Volume Index; BMI, Body mass index; BRI, Body Roundness Index; BAI, Body Adiposity Index; C-index, Conicity Index; LAP, Lipid Accumulation Product; RFM, Relative Fat Mass; VAI, Visceral Adiposity Index; WHR, Waist-to-Hip Ratio; WC, waist circumference; WWI, Weight-adjusted Waist Index; WHtR, Waist-to-Height Ratio.

Supplementary Table 9. Predictive efficacy of obesity-related indices for mortality outcomes in CKM stages 3-4 patients

|  | **All-cause death** | | | **Cardiovascular death** | | | | **Non cardiovascular death** | | | |
| --- | --- | --- | --- | --- | --- | --- | --- | --- | --- | --- | --- |
|  | **AUC (95 CI)** | **P** |  | | **AUC (95 CI)** | **P** |  | | **AUC (95 CI)** | **P** |  |
| **ABSI** | 0.58 (0.57, 0.60) | *Reference* |  | | 0.55 (0.54, 0.57) | *Reference* |  | | 0.56 (0.55, 0.58) | *Reference* |  |
| **BMI** | 0.59 (0.57, 0.60) | 0.705 |  | | 0.54 (0.52, 0.56) | 0.243 |  | | 0.57 (0.56, 0.59) | 0.212 |  |
| **WC** | 0.55 (0.53, 0.56) | 0.002 |  | | 0.52 (0.50, 0.54) | 0.014 |  | | 0.54 (0.53, 0.56) | 0.136 |  |
| **BRI** | 0.55 (0.53, 0.56) | 0.001 |  | | 0.52 (0.50, 0.53) | 0.012 |  | | 0.54 (0.53, 0.56) | 0.075 |  |
| **WWI** | 0.52 (0.51, 0.54) | < 0.001 |  | | 0.52 (0.50, 0.54) | < 0.001 |  | | 0.51 (0.50, 0.53) | 0.005 |  |
| **RFM** | 0.54 (0.53, 0.55) | < 0.001 |  | | 0.52 (0.50, 0.54) | 0.017 |  | | 0.53 (0.52, 0.55) | < 0.001 |  |
| **WHtR** | 0.55 (0.53, 0.56) | < 0.001 |  | | 0.51 (0.49, 0.53) | 0.008 |  | | 0.54 (0.53, 0.56) | 0.106 |  |
| **C-index** | 0.53 (0.51, 0.54) | < 0.001 |  | | 0.52 (0.50, 0.54) | < 0.001 |  | | 0.52 (0.50, 0.53) | < 0.001 |  |
| **VAI** | 0.53 (0.51, 0.55) | < 0.001 |  | | 0.52 (0.50, 0.55) | 0.230 |  | | 0.52 (0.50, 0.54) | < 0.001 |  |
| **LAP** | 0.54 (0.52,0. 56) | 0.013 |  | | 0.51 (0.48, 0.54) | 0.077 |  | | 0.54 (0.52, 0.56) | 0.035 |  |
| **WHR** | 0.53 (0.44, 0.62) | 0.421 |  | | 0.47 (0.31, 0.63) | 0.047 |  | | 0.55 (0.45, 0.66) | 0.964 |  |
| **BAI** | 0.52 (0.43, 0.61) | 0.226 |  | | 0.58 (0.39, 0.76) | 0.450 |  | | 0.50 (0.39, 0.60) | 0.368 |  |
| **AVI** | 0.57 (0.48, 0.66) | 0.704 |  | | 0.45 (0.28, 0.62) | 0.257 |  | | 0.58 (0.47, 0.68) | 0.791 |  |

Abbreviations: ABSI, A Body Shape Index; AVI, Abdominal Volume Index; BMI, Body mass index; BRI, Body Roundness Index; BAI, Body Adiposity Index; C-index, Conicity Index; LAP, Lipid Accumulation Product; RFM, Relative Fat Mass; VAI, Visceral Adiposity Index; WHR, Waist-to-Hip Ratio; WC, waist circumference; WWI, Weight-adjusted Waist Index; WHtR, Waist-to-Height Ratio.

Supplementary Table 10. Predictive efficacy of obesity-related indices for CKM progression in all CKM patients

|  | **CKM stage 1-2→3-4** | |
| --- | --- | --- |
|  | **AUC (95% CI)** | ***P*** |
| **ABSI** | 0.73 (0.72, 0.74) | *Reference* |
| **BMI** | 0.52 (0.51, 0.52) | < 0.001 |
| **WC** | 0.56 (0.55, 0.57) | < 0.001 |
| **BRI** | 0.55 (0.54, 0.56) | < 0.001 |
| **WWI** | 0.70 (0.69, 0.70) | < 0.001 |
| **RFM** | 0.48 (0.47, 0.49) | < 0.001 |
| **WHtR** | 0.58 (0.57, 0.59) | < 0.001 |
| **C-index** | 0.69 (0.69, 0.70) | < 0.001 |
| **VAI** | 0.55 (0.54, 0.56) | < 0.001 |
| **LAP** | 0.56 (0.55, 0.57) | < 0.001 |
| **WHR** | 0.64 (0.62, 0.66) | < 0.001 |
| **BAI** | 0.51 (0.49, 0.53) | < 0.001 |
| **AVI** | 0.55 (0.53, 0.57) | < 0.001 |

Abbreviations: CKM, cardiovascular-kidney-metabolic syndrome; AUC, Area Under Curve; ABSI, A Body Shape Index; AVI, Abdominal Volume Index; BMI, Body mass index; BRI, Body Roundness Index; BAI, Body Adiposity Index; C-index, Conicity Index; LAP, Lipid Accumulation Product; RFM, Relative Fat Mass; VAI, Visceral Adiposity Index; WHR, Waist-to-Hip Ratio; WC, waist circumference; WWI, Weight-adjusted Waist Index; WHtR, Waist-to-Height Ratio

Supplementary Table 11. Sensitivity analysis of obesity-related indices and mortality outcomes in CKM stage 1-2 patients after adjusting other confounding factors

|  | **All-cause** **mortality** | | | **Cardiovascular mortality** | | | | **Non cardiovascular mortality** | | | |
| --- | --- | --- | --- | --- | --- | --- | --- | --- | --- | --- | --- |
|  | **HR (95% CI)** | ***P*** |  | | **HR (95% CI)** | ***P*** |  | | **HR (95% CI)** | ***P*** |  |
| **BMI** |  |  |  | |  |  |  | |  |  |  |
| Q1 | *Reference* |  |  | | *Reference* |  |  | | *Reference* |  |  |
| Q2 | 0.80 (0.74, 0.86) | < 0.001 |  | | 1.06 (0.79, 1.42) | 0.699 |  | | 0.71 (0.62, 0.82) | < 0.001 |  |
| Q3 | 0.77 (0.72, 0.84) | < 0.001 |  | | 1.01 (0.75, 1.36) | 0.925 |  | | 0.75 (0.65, 0.86) | < 0.001 |  |
| Q4 | 0.80 (0.74, 0.87) | 0.008 |  | | 1.33 (0.99, 1.79) | 0.055 |  | | 0.76 (0.66, 0.87) | < 0.001 |  |
| **WC** |  |  |  | |  |  |  | |  |  |  |
| Q1 | *Reference* |  |  | | *Reference* |  |  | | *Reference* |  |  |
| Q2 | 0.83 (0.77, 0.90) | 0.017 |  | | 0.87 (0.64, 1.17) | 0.368 |  | | 0.85 (0.74, 0.98) | 0.027 |  |
| Q3 | 0.76 (0.77, 0.91) | 0.003 |  | | 0.95 (0.70, 1.29) | 0.758 |  | | 0.78 (0.68, 0.91) | 0.002 |  |
| Q4 | 0.94 (0.82, 1.07) | 0.333 |  | | 1.31 (0.98, 1.75) | 0.066 |  | | 0.85 (0.73, 0.99) | 0.039 |  |
| **BRI** |  |  |  | |  |  |  | |  |  |  |
| Q1 | *Reference* |  |  | | *Reference* |  |  | | *Reference* |  |  |
| Q2 | 0.80 (0.70, 0.92) | < 0.001 |  | | 0.94 (0.69, 1.27) | 0.696 |  | | 0.77 (0.67, 0.89) | < 0.001 |  |
| Q3 | 0.80 (0.70, 0.91) | 0.001 |  | | 0.86 (0.63, 1.19) | 0.377 |  | | 0.78 (0.67, 0.91) | 0.002 |  |
| Q4 | 0.91 (0.80, 1.05) | 0.189 |  | | 1.35 (1.01, 1.82) | 0.049 |  | | 0.82 (0.70, 0.95) | 0.011 |  |
| **WWI** |  |  |  | |  |  |  | |  |  |  |
| Q1 | *Reference* |  |  | | *Reference* |  |  | | *Reference* |  |  |
| Q2 | 0.94 (0.82, 1.08) | 0.382 |  | | 0.89 (0.65, 1.23) | 0.490 |  | | 0.95 (0.81, 1.11) | 0.504 |  |
| Q3 | 1.12 (0.97, 1.28) | 0.117 |  | | 1.39 (1.02, 1.89) | 0.034 |  | | 1.05 (0.90, 1.23) | 0.510 |  |
| Q4 | 1.18 (1.02, 1.37) | 0.027 |  | | 1.43 (1.03, 1.99) | 0.033 |  | | 1.12 (0.95, 1.33) | 0.158 |  |
| **ABSI** |  |  |  | |  |  |  | |  |  |  |
| Q1 | *Reference* |  |  | | *Reference* |  |  | | *Reference* |  |  |
| Q2 | 1.02 (0.87, 1.18) | 0.816 |  | | 1.09 (0.78, 1.53) | 0.580 |  | | 0.99 (0.84, 1.18) | 0.980 |  |
| Q3 | 1.17 (1.01, 1.35) | 0.036 |  | | 1.17 (0.84, 1.62) | 0.352 |  | | 1.16 (0.99, 1.37) | 0.064 |  |
| Q4 | 1.41 (1.21, 1.63) | < 0.001 |  | | 1.60 (1.15, 2.22) | 0.005 |  | | 1.35 (1.15, 1.61) | < 0.001 |  |
| **RFM** |  |  |  | |  |  |  | |  |  |  |
| Q1 | *Reference* |  |  | | *Reference* |  |  | | *Reference* |  |  |
| Q2 | 0.95 (0.83, 1.08) | 0.442 |  | | 1.12 (0.84, 1.49) | 0.452 |  | | 0.91 (0.78, 1.05) | 0.194 |  |
| Q3 | 0.93 (0.77, 1.13) | 0.462 |  | | 1.43 (0.96, 2.13) | 0.076 |  | | 0.82 (0.66, 1.02) | 0.077 |  |
| Q4 | 0.92 (0.74, 1.15) | 0.473 |  | | 1.61 (0.99, 2.61) | 0.053 |  | | 0.79 (0.62, 1.02) | 0.070 |  |
| **WHtR** |  |  |  | |  |  |  | |  |  |  |
| Q1 | *Reference* |  |  | | *Reference* |  |  | | *Reference* |  |  |
| Q2 | 0.83 (0.73, 0.94) | 0.005 |  | | 1.10 (0.81, 1.49) | 0.539 |  | | 0.78 (0.67, 0.90) | < 0.001 |  |
| Q3 | 0.91 (0.80, 1.03) | 0.145 |  | | 1.22 (0.90, 1.65) | 0.198 |  | | 0.85 (0.74, 0.98) | 0.027 |  |
| Q4 | 0.97 (0.85, 1.11) | 0.678 |  | | 1.47 (1.09, 2.00) | 0.012 |  | | 0.87 (0.75, 1.02) | 0.090 |  |
| **C-index** |  |  |  | |  |  |  | |  |  |  |
| Q1 | *Reference* |  |  | | *Reference* |  |  | | *Reference* |  |  |
| Q2 | 0.86 (0.77, 1.02) | 0.091 |  | | 0.93 (0.66, 1.28) | 0.642 |  | | 0.87 (0.75, 1.02) | 0.096 |  |
| Q3 | 0.98 (0.86, 1.13) | 0.866 |  | | 1.11 (0.82, 1.52) | 0.493 |  | | 0.95 (0.82, 1.12) | 0.587 |  |
| Q4 | 1.14 (0.99, 1.31) | 0.069 |  | | 1.51 (1.11, 2.06) | 0.009 |  | | 1.06 (0.90, 1.23) | 0.485 |  |

Cox proportional hazards model: adjusted age, sex, race and ethnicity, poverty income ratio, marital states, education, smoking status, alcohol consumption, physical activity, cardiovascular disease, hypertensive, diabetes, chronic kidney disease, stroke, frailty score, antidiabetic medication, antihypertensive medication, lipid-lowering medication.

Abbreviations: HR, hazard ratio; CI, confidence interval; CKM, cardiovascular-kidney-metabolic syndrome; ABSI, A Body Shape Index; BMI, Body mass index; BRI, Body Roundness Index; C-index, Conicity Index; RFM, Relative Fat Mass; WC, waist circumference; WWI, Weight-adjusted Waist Index; WHtR, Waist-to-Height Ratio.

Supplementary Table 12. Sensitivity analysis of obesity-related indices and mortality outcomes in CKM stage 3-4 patients after adjusting other confounding factors

|  | **All-cause mortality** | | | **Cardiovascular mortality** | | | | **Non cardiovascular mortality** | | | |
| --- | --- | --- | --- | --- | --- | --- | --- | --- | --- | --- | --- |
|  | **HR (95% CI)** | ***P*** |  | | **HR (95% CI)** | ***P*** |  | | **HR (95% CI)** | ***P*** |  |
| **BMI** |  |  |  | |  |  |  | |  |  |  |
| Q1 | *Reference* |  |  | | *Reference* |  |  | | *Reference* |  |  |
| Q2 | 0.83 (0.75, 0.91) | < 0.001 |  | | 0.90 (0.76, 1.07) | 0.244 |  | | 0.80 (0.72, 0.89) | < 0.001 |  |
| Q3 | 0.77 (0.70, 0.86) | < 0.001 |  | | 0.82 (0.68, 0.97) | 0.026 |  | | 0.75 (0.66, 0.84) | < 0.001 |  |
| Q4 | 0.78 (0.70, 0.87) | < 0.001 |  | | 0.84 (0.68, 1.03) | 0.097 |  | | 0.76 (0.66, 0.87) | < 0.001 |  |
| **WC** |  |  |  | |  |  |  | |  |  |  |
| Q1 | *Reference* |  |  | | *Reference* |  |  | | *Reference* |  |  |
| Q2 | 0.82 (0.74, 0.91) | < 0.001 |  | | 0.87 (0.72, 1.06) | 0.162 |  | | 0.80 (0.71, 0.90) | < 0.001 |  |
| Q3 | 0.73 (0.66, 0.81) | < 0.001 |  | | 0.85 (0.70, 1.03) | 0.097 |  | | 0.69 (0.60, 0.77) | < 0.001 |  |
| Q4 | 0.78 (0.71, 0.87) | < 0.001 |  | | 0.85 (0.68, 1.02) | 0.079 |  | | 0.77 (0.67, 0.87) | < 0.001 |  |
| **BRI** |  |  |  | |  |  |  | |  |  |  |
| Q1 | *Reference* |  |  | | *Reference* |  |  | | *Reference* |  |  |
| Q2 | 0.75 (0.67, 0.83) | < 0.001 |  | | 0.81 (0.67, 0.98) | 0.034 |  | | 0.73 (0.64, 0.82) | < 0.001 |  |
| Q3 | 0.69 (0.64, 0.77) | < 0.001 |  | | 0.78 (0.65, 0.95) | 0.013 |  | | 0.66 (0.58, 0.75) | < 0.001 |  |
| Q4 | 0.76 (0.67, 0.85) | < 0.001 |  | | 0.80 (0.65, 0.99) | 0.043 |  | | 0.74 (0.65, 0.85) | < 0.001 |  |
| **WWI** |  |  |  | |  |  |  | |  |  |  |
| Q1 | *Reference* |  |  | | *Reference* |  |  | | *Reference* |  |  |
| Q2 | 0.94 (0.82, 1.09) | 0.418 |  | | 1.28 (0.96, 1.71) | 0.082 |  | | 0.83 (0.71, 0.98) | 0.035 |  |
| Q3 | 0.90 (0.79, 1.04) | 0.167 |  | | 1.17 (0.89, 1.54) | 0.249 |  | | 0.82 (0.70, 0.96) | 0.018 |  |
| Q4 | 0.93 (0.81, 1.06) | 0.273 |  | | 1.22 (0.93, 1.60) | 0.144 |  | | 0.83 (0.72, 0.97) | 0.025 |  |
| **ABSI** |  |  |  | |  |  |  | |  |  |  |
| Q1 | *Reference* |  |  | | *Reference* |  |  | | *Reference* |  |  |
| Q2 | 0.98 (0.84, 1.17) | 0.899 |  | | 1.21 (0.88, 1.66) | 0.232 |  | | 0.91 (0.75, .11) | 0.364 |  |
| Q3 | 1.04 (0.88, 1.21) | 0.661 |  | | 1.31 (0.97, 1.76) | 0.072 |  | | 0.94 (0.78, 1.12) | 0.504 |  |
| Q4 | 1.14 (0.98, 1.32) | 0.080 |  | | 1.37 (1.02, 1.83) | 0.034 |  | | 1.07 (0.89, 1.26) | 0.469 |  |
| **RFM** |  |  |  | |  |  |  | |  |  |  |
| Q1 | *Reference* |  |  | | *Reference* |  |  | | *Reference* |  |  |
| Q2 | 0.94 (0.86, 1.04) | 0.266 |  | | 1.04 (0.88, 1.24) | 0.618 |  | | 0.98 (0.81, 1.02) | 0.099 |  |
| Q3 | 0.93 (0.80, 1.07) | 0.334 |  | | 0.92 (0.71, 1.19) | 0.531 |  | | 0.94 (0.79, 1.11) | 0.464 |  |
| Q4 | 0.70 (0.59, 0.84) | < 0.001 |  | | 0.75 (0.54, 1.03) | 0.079 |  | | 0.68 (0.55, 0.85) | < 0.001 |  |
| **WHtR** |  |  |  | |  |  |  | |  |  |  |
| Q1 | *Reference* |  |  | | *Reference* |  |  | | *Reference* |  |  |
| Q2 | 0.79 (0.71, 0.88) | < 0.001 |  | | 0.90 (0.74, 1.09) | 0.310 |  | | 0.75 (0.66, 0.85) | < 0.001 |  |
| Q3 | 0.77 (0.69, 0.85) | < 0.001 |  | | 0.85 (0.71, 1.04) | 0.121 |  | | 0.74 (0.65, 0.84) | < 0.001 |  |
| Q4 | 0.76 (0.68, 0.86) | < 0.001 |  | | 0.88 (0.72, 1.07) | 0.214 |  | | 0.72 (0.64, 0.83) | < 0.001 |  |
| **C-index** |  |  |  | |  |  |  | |  |  |  |
| Q1 | *Reference* |  |  | | *Reference* |  |  | | *Reference* |  |  |
| Q2 | 0.85 (0.74, 0.97) | 0.019 |  | | 0.99 (0.76, 1.29) | 0.964 |  | | 0.79 (0.67, 0.93) | 0.006 |  |
| Q3 | 0.83 (0.73, 0.95) | 0.006 |  | | 1.09 (0.85, 1.40) | 0.452 |  | | 0.74 (0.64, 0.86) | < 0.001 |  |
| Q4 | 0.87 (0.76, 0.98) | 0.028 |  | | 0.99 (0.78, 1.25) | 0.967 |  | | 0.83 (0.72, 0.96) | 0.011 |  |

Cox proportional hazards model: adjusted age, sex, race and ethnicity, poverty income ratio, marital states, education, smoking status, alcohol consumption, physical activity, cardiovascular disease, hypertensive, diabetes, chronic kidney disease, stroke, frailty score, antidiabetic medication, antihypertensive medication, lipid-lowering medication.

Abbreviations: HR, hazard ratio; CI, confidence interval; CKM, cardiovascular-kidney-metabolic syndrome; ABSI, A Body Shape Index; BMI, Body mass index; BRI, Body Roundness Index; C-index, Conicity Index; RFM, Relative Fat Mass; WC, waist circumference; WWI, Weight-adjusted Waist Index; WHtR, Waist-to-Height Ratio.

Supplementary Table 13. Sensitivity analysis of obesity-related indices and mortality outcomes in CKM stage 3-4 patients after excluding patients who died within two years

|  | **All-cause mortality** | | | **Cardiovascular mortality** | | | | **Non cardiovascular mortality** | | | |
| --- | --- | --- | --- | --- | --- | --- | --- | --- | --- | --- | --- |
|  | **HR (95% CI)** | ***P*** |  | | **HR (95% CI)** | ***P*** |  | | **HR (95% CI)** | ***P*** |  |
| **BMI** |  |  |  | |  |  |  | |  |  |  |
| Q1 | *Reference* |  |  | | *Reference* |  |  | | *Reference* |  |  |
| Q2 | 0.85 (0.79, 0.93) | < 0.001 |  | | 0.96 (0.83, 1.12) | 0.623 |  | | 0.79 (0.72, 0.86) | < 0.001 |  |
| Q3 | 0.82 (0.75, 0.89) | < 0.001 |  | | 0.87 (0.75, 1.03) | 0.108 |  | | 0.77 (0.70, 0.85) | < 0.001 |  |
| Q4 | 0.91 (0.83, 0.99) | 0.039 |  | | 1.05 (0.88, 1.24) | 0.595 |  | | 0.83 (0.75, 0.92) | < 0.001 |  |
| **WC** |  |  |  | |  |  |  | |  |  |  |
| Q1 | *Reference* |  |  | | *Reference* |  |  | | *Reference* |  |  |
| Q2 | 0.82 (0.75, 0.89) | < 0.001 |  | | 0.87 (0.74, 1.04) | 0.136 |  | | 0.80 (0.72, 0.88) | < 0.001 |  |
| Q3 | 0.78 (0.72, 0.85) | < 0.001 |  | | 0.90 (0.76, 1.07) | 0.251 |  | | 0.75 (0.67, 0.82) | < 0.001 |  |
| Q4 | 0.92 (0.84, 1.00) | 0049 |  | | 1.01 (0.85, 1.20) | 0.907 |  | | 0.88 (0.80, 0.98) | 0.023 |  |
| **BRI** |  |  |  | |  |  |  | |  |  |  |
| Q1 | *Reference* |  |  | | *Reference* |  |  | | *Reference* |  |  |
| Q2 | 0.77 (0.71, 0.84) | < 0.001 |  | | 0.89 (0.75, 1.06) | 0.205 |  | | 0.74 (0.67, 0.82) | < 0.001 |  |
| Q3 | 0.76 (0.69, 0.83) | < 0.001 |  | | 0.84 (0.71, 1.01) | 0.065 |  | | 0.73 (0.66, 0.81) | < 0.001 |  |
| Q4 | 0.88 (0.81, 0.97) | 0.010 |  | | 1.00 (0.83, 1.19) | 0.996 |  | | 0.85 (0.77, 0.95) | 0.004 |  |
| **WWI** |  |  |  | |  |  |  | |  |  |  |
| Q1 | *Reference* |  |  | | *Reference* |  |  | | *Reference* |  |  |
| Q2 | 0.94 (0.84, 1.04) | 0.231 |  | | 1.09 (0.87, 1.35) | 0.435 |  | | *0.89 (0.79, 1.01)* | 0.076 |  |
| Q3 | 0.99 (0.90, 1.10) | 0.920 |  | | 1.18 (0.96, 1.46) | 0.109 |  | | *0.94 (0.84, 1.06)* | 0.315 |  |
| Q4 | 1.07 (0.96, 1.18) | 0.177 |  | | 1.27 (1.03, 1.57) | 0.022 |  | | 1.02 (0.90, 1.14) | 0.773 |  |
| **ABSI** |  |  |  | |  |  |  | |  |  |  |
| Q1 | *Reference* |  |  | | *Reference* |  |  | | *Reference* |  |  |
| Q2 | 1.03 (0.91, 1.15) | 0.678 |  | | 1.15 (0.91, 1.46) | 0.237 |  | | 0.98 (0.86, 1.13) | 0.863 |  |
| Q3 | 1.11 (0.99, 1.23) | 0.065 |  | | 1.26 (1.01, 1.58) | 0.041 |  | | 1.06 (0.93, 1.20) | 0.337 |  |
| Q4 | 1.28 (1.15, 1.43) | < 0.001 |  | | 1.38 (1.11, 1.72) | 0.004 |  | | 1.26 (1.11, 1.42) | < 0.001 |  |
| **RFM** |  |  |  | |  |  |  | |  |  |  |
| Q1 | *Reference* |  |  | | *Reference* |  |  | | *Reference* |  |  |
| Q2 | 0.96 (0.89, 1.05) | 0.431 |  | | 1.01 (0.86, 1.18) | 0.871 |  | | 0.95 (0.86, 1.05) | 0.323 |  |
| Q3 | 1.00 (0.88, 1.13) | 0.973 |  | | 1.07 (0.85, 1.35) | 0.549 |  | | 0.97 (0.85, 1.13) | 0.773 |  |
| Q4 | 0.92 (0.79, 1.06) | 0.246 |  | | 1.09 (0.82, 1.44) | 0.541 |  | | 0.87 (0.73, 1.03) | 0.097 |  |
| **WHtR** |  |  |  | |  |  |  | |  |  |  |
| Q1 | *Reference* |  |  | | *Reference* |  |  | | *Reference* |  |  |
| Q2 | 0.82 (0.75, 0.90) | < 0.001 |  | | 1.02 (0.85, 1.22) | 0.804 |  | | 0.77 (0.69, 0.85) | < 0.001 |  |
| Q3 | 0.85 (0.78, 0.92) | < 0.001 |  | | 0.97 (0.82, 1.16) | 0.798 |  | | 0.82 (0.74, 0.90) | < 0.001 |  |
| Q4 | 0.92 (0.84, 1.01) | 0.087 |  | | 1.13 (0.95, 1.35) | 0.173 |  | | 0.86 (0.78, 0.96) | 0.006 |  |
| **C-index** |  |  |  | |  |  |  | |  |  |  |
| Q1 | *Reference* |  |  | | *Reference* |  |  | | *Reference* |  |  |
| Q2 | 0.87 (0.78, 0.96) | 0.008 |  | | 0.97 (0.78, 1.21) | 0.805 |  | | 0.84 (0.75, 0.95) | 0.004 |  |
| Q3 | 0.90 (0.82, 0.99) | 0.046 |  | | 1.13 (0.92, 1.38) | 0.232 |  | | 0.84 (0.75, 0.94) | 0.003 |  |
| Q4 | 1.02 (0.93, 1.12) | 0.646 |  | | 1.13 (0.93, 1.38) | 0.210 |  | | 0.99 (0.89, 1.11) | 0.941 |  |

Cox proportional hazards model: adjusted age, sex, race and ethnicity, poverty income ratio, marital states, education, smoking status, alcohol consumption, physical activity, cardiovascular disease, hypertensive, diabetes, chronic kidney disease, stroke.

Abbreviations: HR, hazard ratio; CI, confidence interval; CKM, cardiovascular-kidney-metabolic syndrome; ABSI, A Body Shape Index; BMI, Body mass index; BRI, Body Roundness Index; C-index, Conicity Index; RFM, Relative Fat Mass; WC, waist circumference; WWI, Weight-adjusted Waist Index; WHtR, Waist-to-Height Ratio


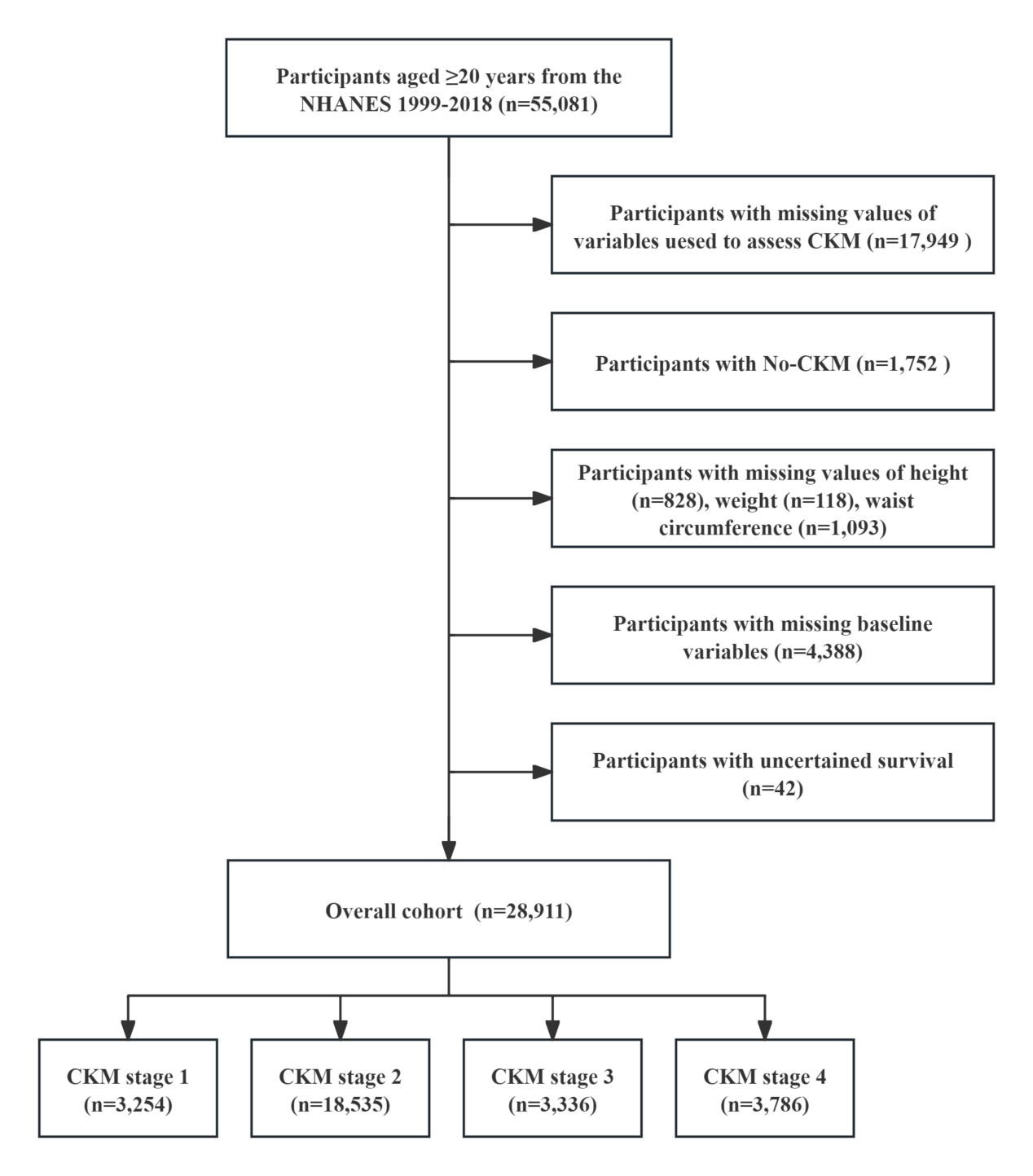


**Supplementary Fig. 1. Flowchart of this study.** CKM, cardiovascular-kidney-metabolic syndrome; NHANES, National Health and Nutrition Examination Survey.

**
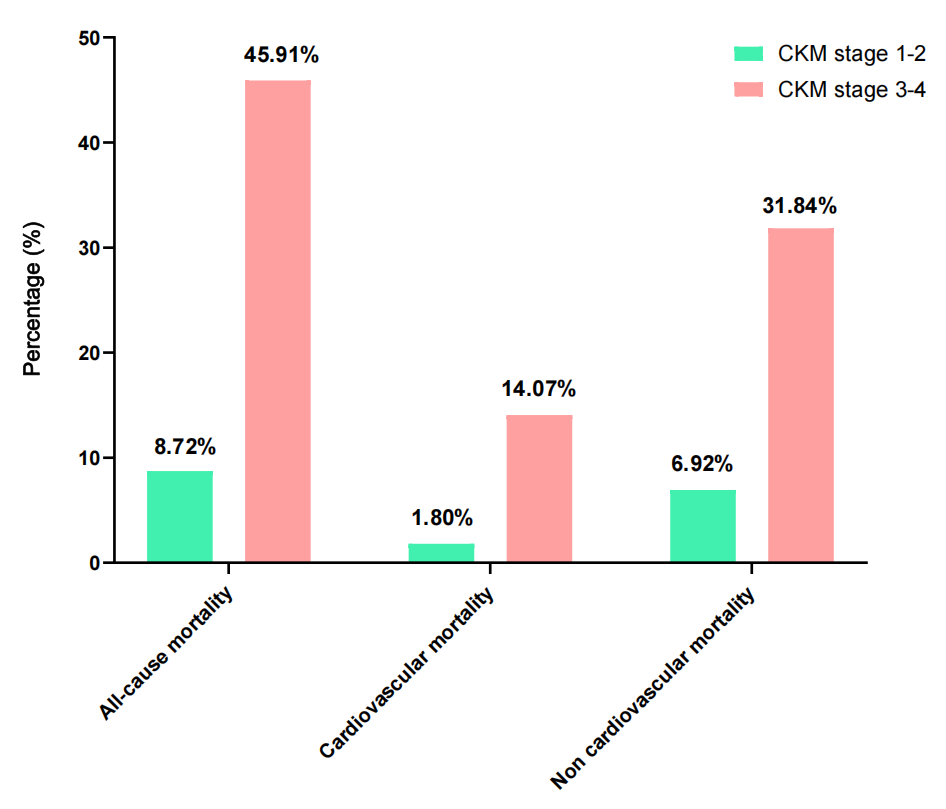
**

**Supplementary Fig. 2. Mortality in different CKM stages.** CKM, cardiovascular-kidney-metabolic syndrome.


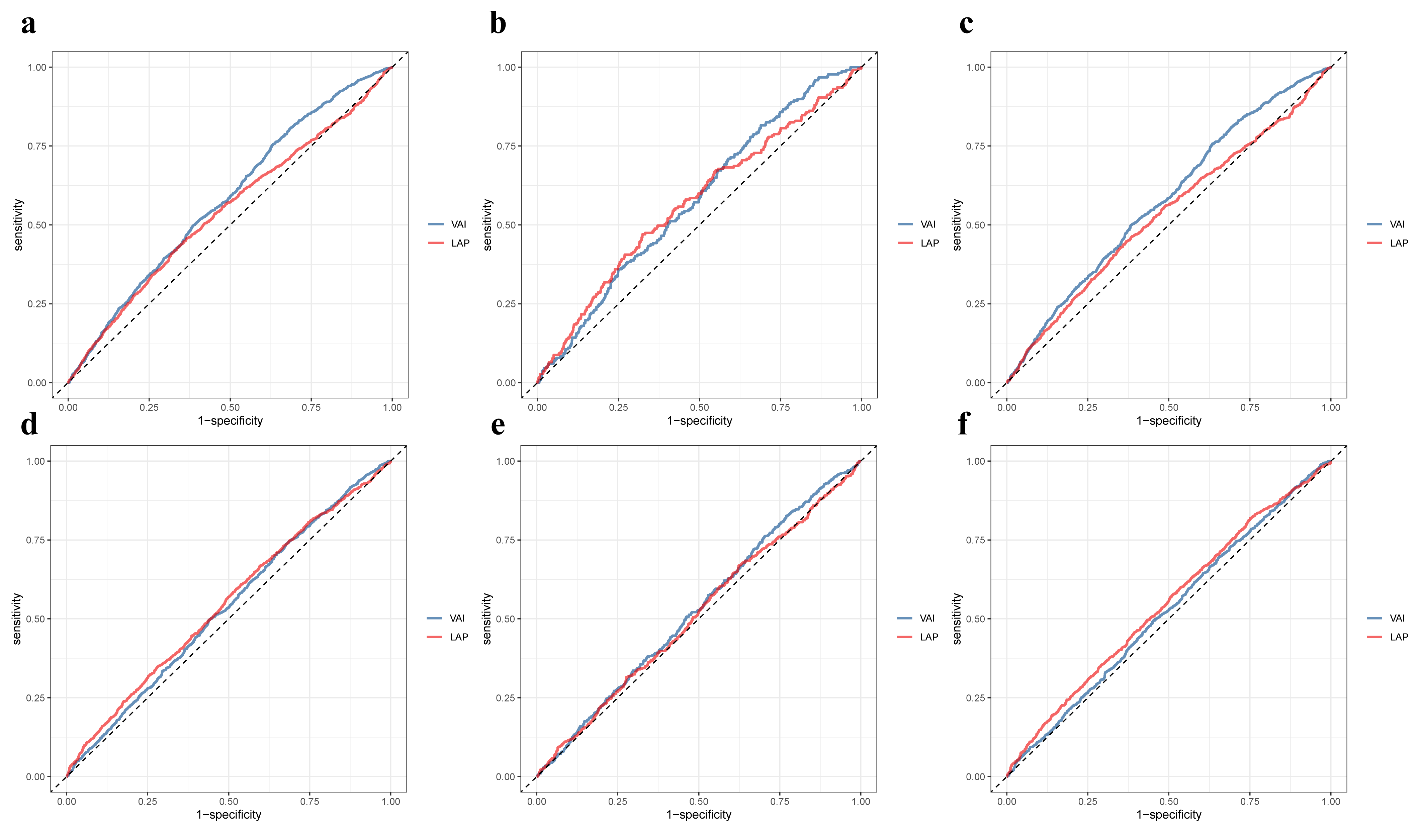


**Supplementary Fig. 3. Receiver Operating Characteristic Crues for Associations Between Obesity-related indices and Mortality Outcomes in CKM stages 1-2 and 3-4 Patients.** (a) all-cause mortality, (b) cardiovascular mortality, (c) non-cardiovascular mortality in CKM stage 1-2 patients; (d) all-cause mortality, (e) cardiovascular mortality, (f) non-cardiovascular mortality in CKM stage 3-4 patients. VAI, Visceral Adiposity Index; LAP, Lipid Accumulation Product.


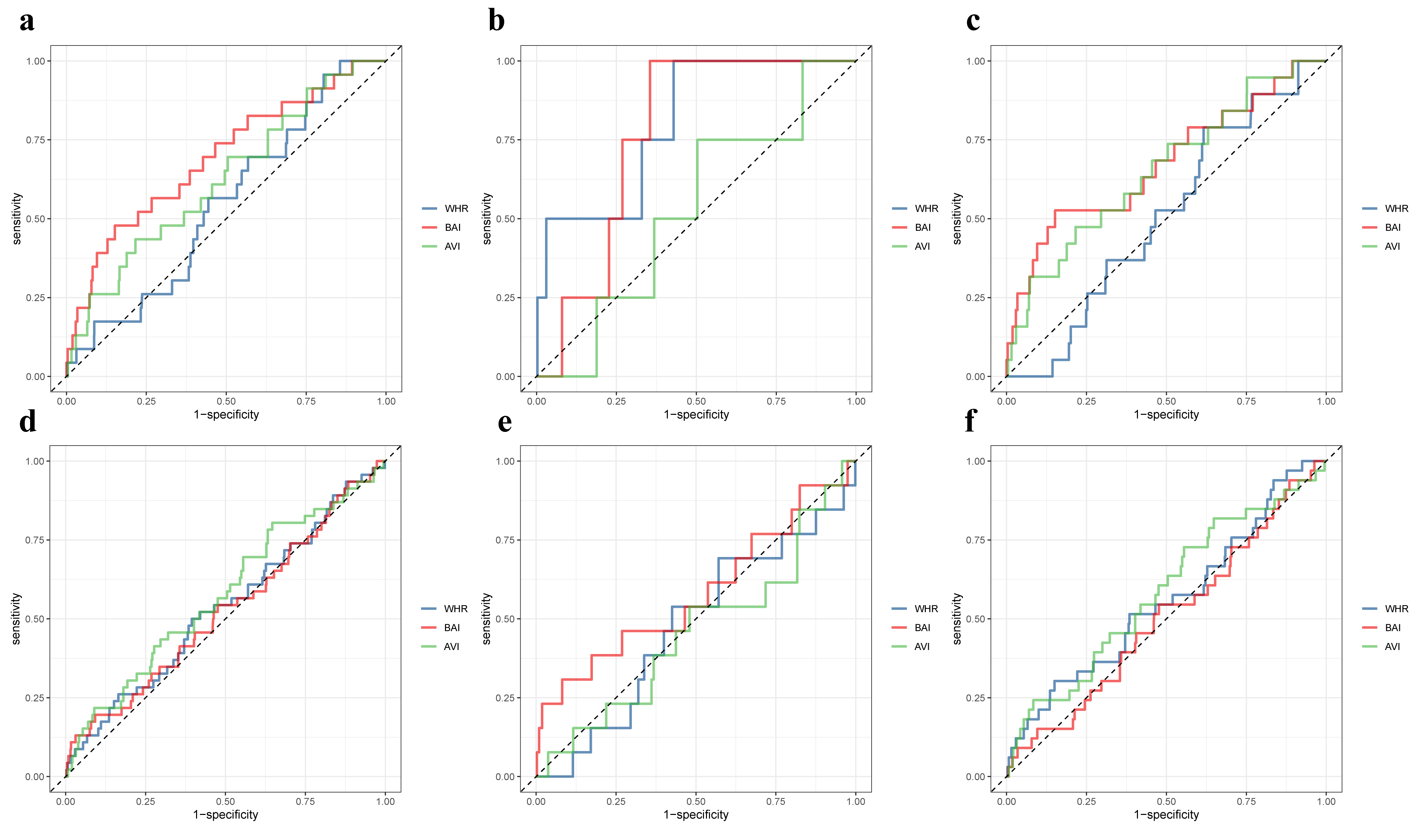


**Supplementary Fig. 4. Receiver Operating Characteristic Crues for Associations Between Obesity-related indices and Mortality Outcomes in CKM stage 1-2 and 3-4 Patients.** (a) all-cause mortality, (b) cardiovascular mortality, (c) non-cardiovascular mortality in CKM stage 1-2 patients, (d) all-cause mortality, (e) cardiovascular mortality, (f) non-cardiovascular mortality in CKM stage 3-4 patients. WHR, Waist-to-Hip Ratio; BAI, Body Adiposity Index; AVI, Abdominal Volume Index.


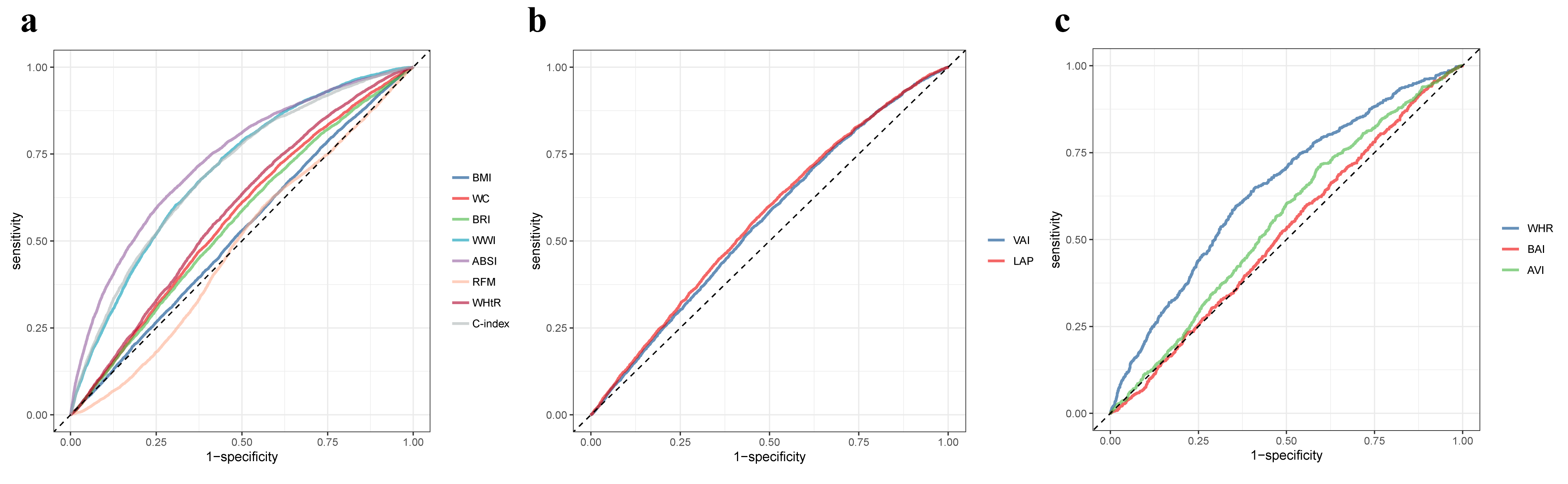


**Supplementary Fig. 5. Receiver Operating Characteristic Crues for Associations Between Obesity-related indices and CKM progression.** ABSI, A Body Shape Index; BMI, Body mass index; BRI, Body Roundness Index; C-index, Conicity Index; RFM, Relative Fat Mass; WC, waist circumference; WWI, Weight-adjusted Waist Index; WHtR, Waist-to-Height Ratio; VAI, Visceral Adiposity Index; LAP, Lipid Accumulation Product; WHR, Waist-to-Hip Ratio; BAI, Body Adiposity Index; AVI, Abdominal Volume Index.
